# Construction and Validation of a Ferroptosis-Related lncRNA Signature as a Novel Biomarker for Prognosis, Immunotherapy and Targeted Therapy in Hepatocellular Carcinoma

**DOI:** 10.3389/fcell.2022.792676

**Published:** 2022-02-22

**Authors:** Ze Zhang, Wenwen Zhang, Yafei Wang, Tao Wan, Bingyang Hu, Chonghui Li, Xinlan Ge, Shichun Lu

**Affiliations:** ^1^ Medical School of Chinese People’s Liberation Army (PLA), Beijing, China; ^2^ Faculty of Hepato-Pancreato-Biliary Surgery, Chinese PLA General Hospital, Beijing, China; ^3^ Institute of Hepatobiliary Surgery of Chinese PLA, Beijing, China; ^4^ Key Laboratory of Digital Hepatobiliary Surgery, PLA, Beijing, China

**Keywords:** hepatocellular carcinoma, ferroptosis, long non-coding RNAs, prognostic, immunotherapy, targeted therapy, TCGA

## Abstract

Recently, immunotherapy combined with targeted therapy has significantly prolonged the survival time and improved the quality of life of patients with hepatocellular carcinoma (HCC). However, HCC treatment remains challenging due to the high heterogeneity of this malignancy. Sorafenib, the first-line drug for the treatment of HCC, can inhibit the progression of HCC by inducing ferroptosis. Ferroptosis is associated with the formation of an immunosuppressive microenvironment in tumours. Moreover, long non-coding RNAs (lncRNAs) are strongly associated with ferroptosis and the progression of HCC. Discovery of ferroptosis-related lncRNAs (FR-lncRNAs) is critical for predicting prognosis and the effectiveness of immunotherapy and targeted therapies to improve the quality and duration of survival of HCC patients. Herein, all cases from The Cancer Genome Atlas (TCGA) database were divided into training and testing groups at a 6:4 ratio to construct and validate the lncRNA signatures. Least Absolute Shrinkage and Selection Operator (LASSO) regression and Cox regression analyses were used to screen the six FR-lncRNAs (including MKLN1-AS, LINC01224, LNCSRLR, LINC01063, PRRT3-AS1, and POLH-AS1). Kaplan–Meier (K–M) and receiver operating characteristic (ROC) curve analyses demonstrated the optimal predictive prognostic ability of the signature. Furthermore, a nomogram indicated favourable discrimination and consistency. For further validation, we used real-time quantitative polymerase chain reaction (qRT-PCR) to analyse the expression of LNCSRLR, LINC01063, PRRT3-AS1, and POLH-AS1 in HCC tissues. Moreover, we determined the ability of the signature to predict the effects of immunotherapy and targeted therapy in patients with HCC. Gene set enrichment analysis (GSEA) and somatic mutation analysis showed that ferroptosis-related pathways, immune-related pathways, and TP53 mutations may be strongly associated with the overall survival (OS) outcomes of HCC patients. Overall, our study suggests that a new risk model of six FR-lncRNAs has a significant prognostic value for HCC and that it could contribute to precise and individualised HCC treatment.

## Introduction

Liver cancer ranks sixth in the world in terms of incidence and is the fourth most common cause of cancer-related deaths (Villanueva, 2019). HCC is the most common malignancy of the liver, accounting for approximately 90% of liver cancer cases (Llovet et al., 2021). In the early stages of HCC, curative resection, transplantation, and local ablation are the preferred treatment options, but most patients with HCC are diagnosed at an advanced stage and cannot be cured using the above approaches (Llovet et al., 2021). Despite the many advances in the treatment of HCC, such as immunotherapy and targeted therapies, the median survival time for patients with advanced HCC is only 11–13 months (Villanueva, 2019). Given the high mortality rate of HCC, it is imperative to further explore biomarkers that can predict the effectiveness of HCC immunotherapy or targeted therapy, discover new prognosis-related biomarkers, and identify new therapeutic targets to prolong the survival time of patients with HCC.

Ferroptosis is a type of regulated cell death which, unlike apoptosis, pyroptosis, and necroptosis, is caused by the accumulation of lipid peroxide (Stockwell et al., 2017). Recent studies have shown that ferroptosis is associated with the progression and treatment response of various types of tumours (Chen et al., 2021a). For instance, ferroptosis can induce inflammation-related immunosuppression in the tumour immune microenvironment (TIME), thereby promoting tumour progression, whereas mutations in TP53 and RAS genes are closely related to ferroptosis (Chen et al., 2021a). However, the regulatory mechanisms of ferroptosis in HCC remain to be investigated and are far from being applied for the treatment of HCC (Nie et al., 2018; Capelletti et al., 2020). Therefore, identifying the main regulators associated with ferroptosis is a crucial way to broaden the therapeutic approach to HCC.

Long non-coding RNAs (LncRNAs) are most commonly defined as RNAs that are more than 200 nucleotides in length and do not encode proteins (Quinn and Chang, 2016). In HCC, lncRNAs are specifically involved in protein synthesis, degradation, and epigenetic regulation (Mai et al., 2019). HCC-related lncRNAs can regulate the protein modifications of transcription factors and influence protein function by regulating the corresponding cellular signalling pathways (Li et al., 2017; Yan et al., 2017; Zhang et al., 2018). Furthermore, recent studies have demonstrated that GABPB1 and its antisense lncRNA GABPB1-AS1 are closely linked to erastin-induced ferroptosis and that GABPB1 and GABPB1-AS1 can be used as treatment targets for HCC patients (Qi et al., 2019). However, the mechanism underlying the role of FR-lncRNAs in HCC is unclear, and its importance in the therapeutic and prognostic value of HCC needs to be further elucidated.

In this study, we identified and validated a prognostic signature based on ferroptosis-related (FR)-lncRNAs. We also explored the association between the risk model and immune cell infiltration, immune function, sensitivity of immune checkpoint inhibitor (ICI) treatment, and sorafenib treatment. Furthermore, we used gene set enrichment analysis (GSEA) to reveal the pathways enriched in the high- and low-risk groups of the signature. Finally, we validated the significantly differentially expressed lncRNAs in this signature between the tumour and para-tumour tissues *in vitro*. In conclusion, this new prognostic signature is more accurate and convenient than previous signatures in predicting the prognosis and treatment outcomes of HCC patients.

## Methods

### Patient Datasets and Processing

**FIGURE 1:**
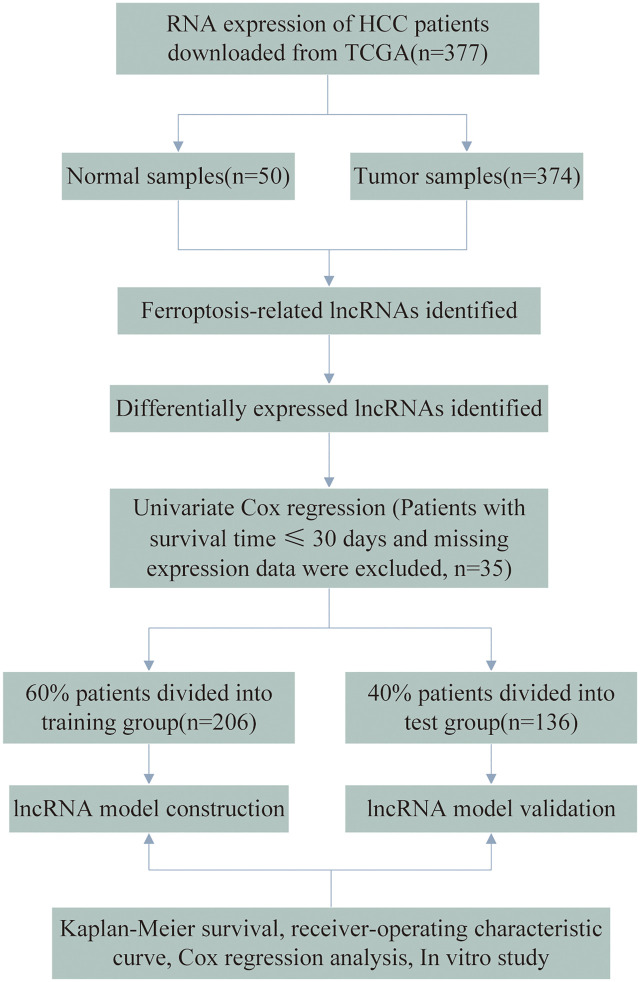
Flow chart showing the process of identifying FR-lncRNAs.

Gene expression profiles and clinical data from patients with HCC were downloaded from The Cancer Genome Atlas (TCGA) database. Considering the likelihood of non-cancer deaths, patients with a survival time of ≤30 days and missing expression data were excluded (*n* = 35), leaving 342 HCC patients in the final cohort. The flow chart of the analysis process is presented in Figure 1. A total of 342 HCC cases were randomly assigned to the training and testing sets at a ratio of 6:4 for systematic analysis employing the R project “caret” package. Both the training and testing sets needed to comply with the following requirements: 1) cases were randomly classified into training and test groups, and 2) the clinical characteristics of the subjects in both groups were similar. Gene transfer format (GTF) files were downloaded from Ensembl for annotation to distinguish messenger RNAs (mRNAs) from lncRNAs for subsequent analysis. The data of ferroptosis genes (FRGs) were downloaded from FerrDb (Zhou and Bao, 2020) and led to the identification of 259 FRGs. (Supplementary Table S1). Correlation analysis was performed between the FRGs and all the lncRNAs. Those with ferroptosis gene correlation coefficients higher than 0.4 and *p* values lesser than 0.001 were considered FR-lncRNAs. To identify the differentially expressed FR-lncRNAs, we used the R package “limma” for differential expression analysis of FR-lncRNAs. Significantly differentially expressed FR-lncRNAs fulfilled the following criteria: *p* < 0.05 and |log2FC| ≥ 1.

### Construction of a Prognostic Risk Signature

We screened lncRNAs associated with OS in patients with HCC using univariate Cox regression analysis. *p* < 0.05 was considered statistically significant. For the training group, LASSO regression analysis was performed using the R project “glmnet” package to further select the screened lncRNAs. To prevent over-fitting, 1,000 rounds of cross-validation were used to adjust the parameter selection, and the partial likelihood deviation met the minimum criteria. We then performed a multivariate Cox regression analysis and identified six FR-lncRNAs. Subsequently, we calculated the six FR-lncRNAs corresponding coefficients to construct a prognostic risk score profile for HCC. The formula was established as follow:

Risk score = coef lncRNA1 × lncRNA1 expression + coef lncRNA2 × lncRNA2 expression + · ··· + coef lncRNA N × lncRNA N expression.

### Confirmation of the lncRNA Signature

Patients were classified into high- or low-risk groups according to the median risk score threshold. The “survival” package in R was used to perform Kaplan–Meier (K–M) survival analysis in both datasets to verify the predictive ability of the signature. Time-dependent ROC curves were constructed, and the area under the time-dependent ROC curve (AUC) values for 1, 3, and 5 years were calculated. Moreover, multivariate Cox regression analysis was conducted to assess whether the risk score model could be used as an independent predictor of OS in HCC patients. The predictive efficacy of this six-FR-lncRNA signature was further confirmed in the testing and whole cohorts.

### Nomogram Construction and Assessment

The R package “rms” and “regplot” were used to construct the nomogram based on the six-FR-lncRNA signature, which integrated the signature, age, and stage. We have estimated the prognosis of HCC patients at 1, 3, and 5 years in the nomogram. Finally, calibration curves were plotted to assess the accuracy and reliability of the nomogram.

### RNA Isolation and qRT-PCR Analysis

Eleven fresh pathologically confirmed HCC tumour samples were paired with their para-tumour tissues for RNA extraction and qRT-PCR. None of these patients had been previously diagnosed with any other type of human cancer and had not received anti-cancer treatment. All specimens were rapidly frozen in liquid nitrogen (−196°C) immediately after tissue excision. Total cellular RNA was extracted using the TRIzol reagent (Invitrogen, California, United States). Complementary DNA (cDNA) was synthesised using the PrimeScript Reverse Transcriptase Reagent Kit (Takara Bio, Inc., Japan). Amplification and detection were performed using TB Green^®^ Premix Ex Taq™ II (Takara, Tokyo, Japan) in the ABI Step One Plus Real-Time PCR system (Applied Biosystems). β-Actin was used as an endogenous control. The expression level of PRRT3-AS1, LNCSRLR, LINC01063, and POLH-AS1 was normalised to that of β-actin using the 2^−ΔΔCt^ method. The primer sequences are listed in Supplementary Table S2. This study was approved by the ethics committee of the Chinese PLA General Hospital (approval no. S2018-111-01). Written informed consent was obtained from all patients.

### Estimation of the Immune Cell Types and Analysis of Tumour Immune Infiltration Cell Types

Quantitative translation of the tumour tissue transcriptome data into the absolute abundance of immune and stromal cells by Cell-type Identification By Estimating Relative Subsets Of RNA Transcripts (CIBERSORT) analysis was conducted to assess the proportion of 22 human immune cell subpopulations (Newman et al., 2015; Becht et al., 2016). Differences in each type of immune cell were compared between the high-risk and low-risk groups to assess differences in the tumour immune microenvironment between the two groups. Then, we implemented a single sample GSEA (ssGSEA) approach using the gene set variation analysis (GSVA) and “GSEABase” packages to analyse the immune functions and inflammatory infiltration profiles (Newman et al., 2019). Immune infiltrating cells and functions were compared between the high- and low-risk groups using the Wilcoxon test.

### Analysis of the Expression of Immune Checkpoint Genes and Sensitivity to Clinical Treatment in the High- and Low-Risk Groups

We analysed the expression of 47 immune checkpoint genes between the high- and low-risk groups. Then, we used the tumour immune dysfunction and exclusion (TIDE) algorithm to predict the effectiveness of immunotherapy (Jiang et al., 2018). We also calculated the half-inhibitory concentration (IC50) of sorafenib in the entire dataset. The difference in the IC50 of sorafenib between the two groups was compared using Wilcoxon signed-rank test and the results were obtained by using the R packages “pRRophetic” and “ggplot2.”

### Somatic Variant Analysis

Gene somatic mutation data based on the whole-exome sequencing platform of TCGA-Liver Hepatocellular Carcinoma (LIHC) datasets were downloaded from the Genomic Data Commons (GDC) database on August 22, 2021. The downloaded Mutation Annotation Format (MAF) files of simple nucleotide variation (workflow type: varScan2 variant aggregation and masking) were analysed using the R package “maftools.”

### Construction of the lncRNA-FRG Co-Expression Network, Copy Number Variations of FRGs Associated With the Signature, and Functional Analysis

According to the results of the previous co-expression analysis, Cytoscape (version 3.8.2) was used to visualise the co-expression network of prognostic FR-lncRNAs and FRGs. CNV data were obtained through the UCSC Xena program, which is publicly available under specific guidelines. The landscape of genomic CNV in chromosomes was plotted using the “RCircos” R package. GSEA software, version 4.1.0 was used to reveal the signal transduction pathways (Subramanian et al., 2005). The gene sets used in this work were c2.cp.kegg.v7.4.symbols.gmt (Kanehisa et al., 2017) and h.all.v7.4.symbols.gmt (Subramanian et al., 2005). A nominal *p*-value of <0.05 was used as the screening criterion.

### Statistical Analysis

We used R version 4.1.0 (Institute for Statistics and Mathematics, Vienna, Austria), GraphPad Prism 8 (GraphPad Software Inc., La Jolla, CA, United States), and SPSS 25.0 (SPSS, IL, United States) to analyse our data. Survival analysis was performed using K–M and log-rank tests. The *t*-test or Mann–Whitney *U* test was used to compare two independent groups. Categorical data were analysed using the χ^2^ test. TIDE scores between the high-risk group and low-risk group were compared using the Wilcoxon test. *p* < 0.05 was considered statistically significant (*p < 0.05, **p < 0.01, ***p < 0.001).

## Results

### Screening of FR-lncRNAs and Differential Expression Analysis

**FIGURE 2:**
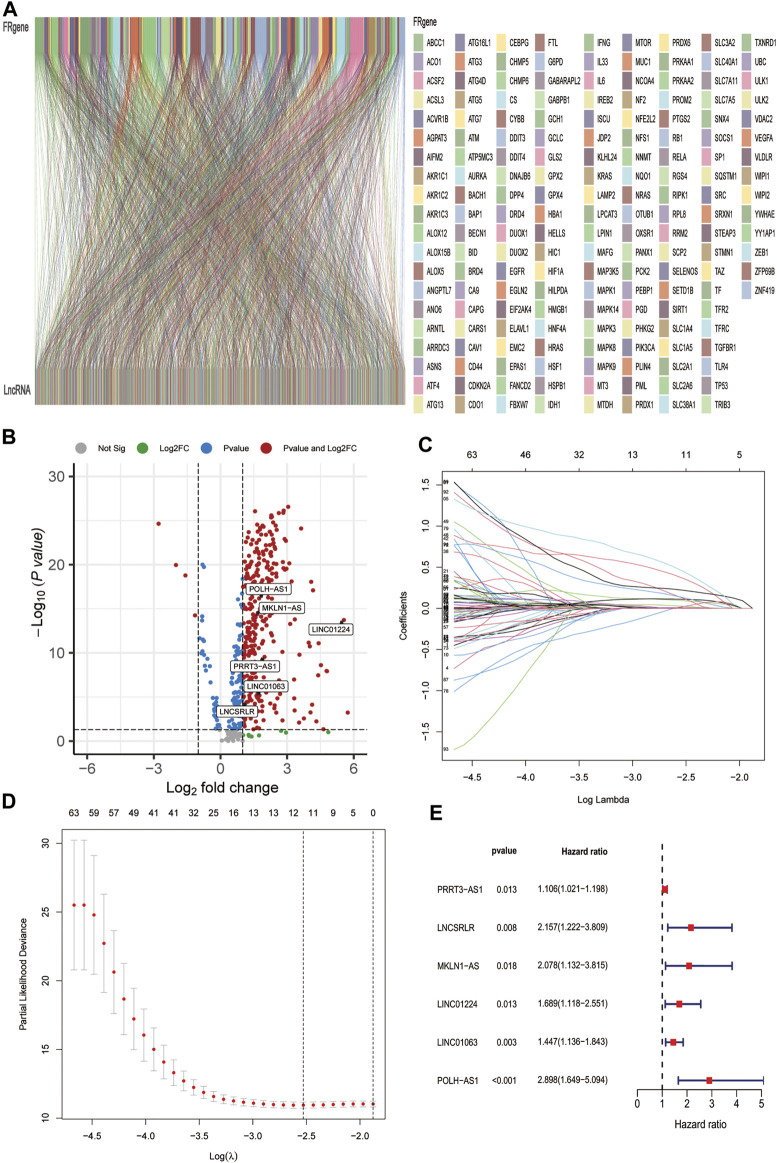
Identification of FR-related lncRNAs in HCC patients and construction of the ferroptosis-related lncRNA prognostic signature. **(A)** Sankey relational diagram for 269 ferroptosis genes and 496 FR-related lncRNAs. **(B)** Volcano plot of the differentially expressed FR-lncRNAs **(C)** LASSO coefficient profiles of 115 FR-lncRNAs. **(D)** To construct a prognostic signature, 11 best candidate FR-lncRNAs were obtained. **(E)** Forest map showed 6 lncRNAs identified by multivariate Cox regression in the training group.

The data of 374 HCC samples and 50 normal samples were downloaded from the LIHC project of TCGA. Then, data annotation was performed according to the GTF file from Ensembl and divided into lncRNA and mRNA data, followed by FRG and lncRNA co-expression analysis and FR-lncRNA co-expression network visualization using the Sankey diagram in Figure 2A. Finally, 548 FR-lncRNAs were identified totally (|r | > 0.4, *p* < 0.001), of which 336 were identified as differentially expressed FR-lncRNAs according to the standard *p* < 0.05, and |log2FC| > 1 (Figure 2B). Among these differentially expressed FR-lncRNAs, 332 were upregulated and four were downregulated.

### Construction and Confirmation of the FR-lncRNAs Signature

A total of 342 HCC patients were included according to the inclusion criteria. We performed univariate Cox regression analysis and screened 115 differentially expressed FR-lncRNAs associated with OS (*p* < 0.05) (Supplementary Table S3). The 342 HCC patients (entire dataset) were assigned to the training and testing groups at a ratio of 6:4 randomly, and the clinical features were similar between the three groups (Table 1). Then, LASSO regression and Cox proportional hazard model analyses were applied to identify the best model in the training dataset (Figures 2C,D). Furthermore, multivariate analysis identified six FR-lncRNAs risk scores for HCC (Supplementary Table S4), according to the following formula:

Riskscore = PRRT3-AS1 * 0.101 + LNCSRLR * 0.769 + MKLN1-AS * 0.732 + LINC01224 * 0.524 + LINC01063 * 0.370 + POLH-AS1 * 1.06

**TABLE 1:** Clinical characteristics of 342 patients with hepatocellular carcinoma.

Character	Training dataset	Test dataset	Entire dataset	*p-*value
	*n* = 206	*n* = 136	*n* = 342	
Age				0.803
≤65	133	83	216	
>65	73	53	126	
Gender				0.544
Female	61	48	109	
Male	145	88	233	
Grade				0.636
G1-G2	122	92	214	
G3-G4	81	42	123	
Unknow	3	2	5	
TNM stage				0.964
I-II	145	93	238	
III-IV	50	33	83	
Unknow	11	10	21	
Tumor stage				0.996
T1-T2	154	99	252	
T3-T4	51	36	87	
Unknow	2	1	3	

Figure 2E shows the relationship between each lncRNA and OS. The expression of six FR-lncRNAs was significantly upregulated in HCC by comparing HCC and normal tissues in the TCGA transcriptome profiles (Figure 2B).

**FIGURE 3:**
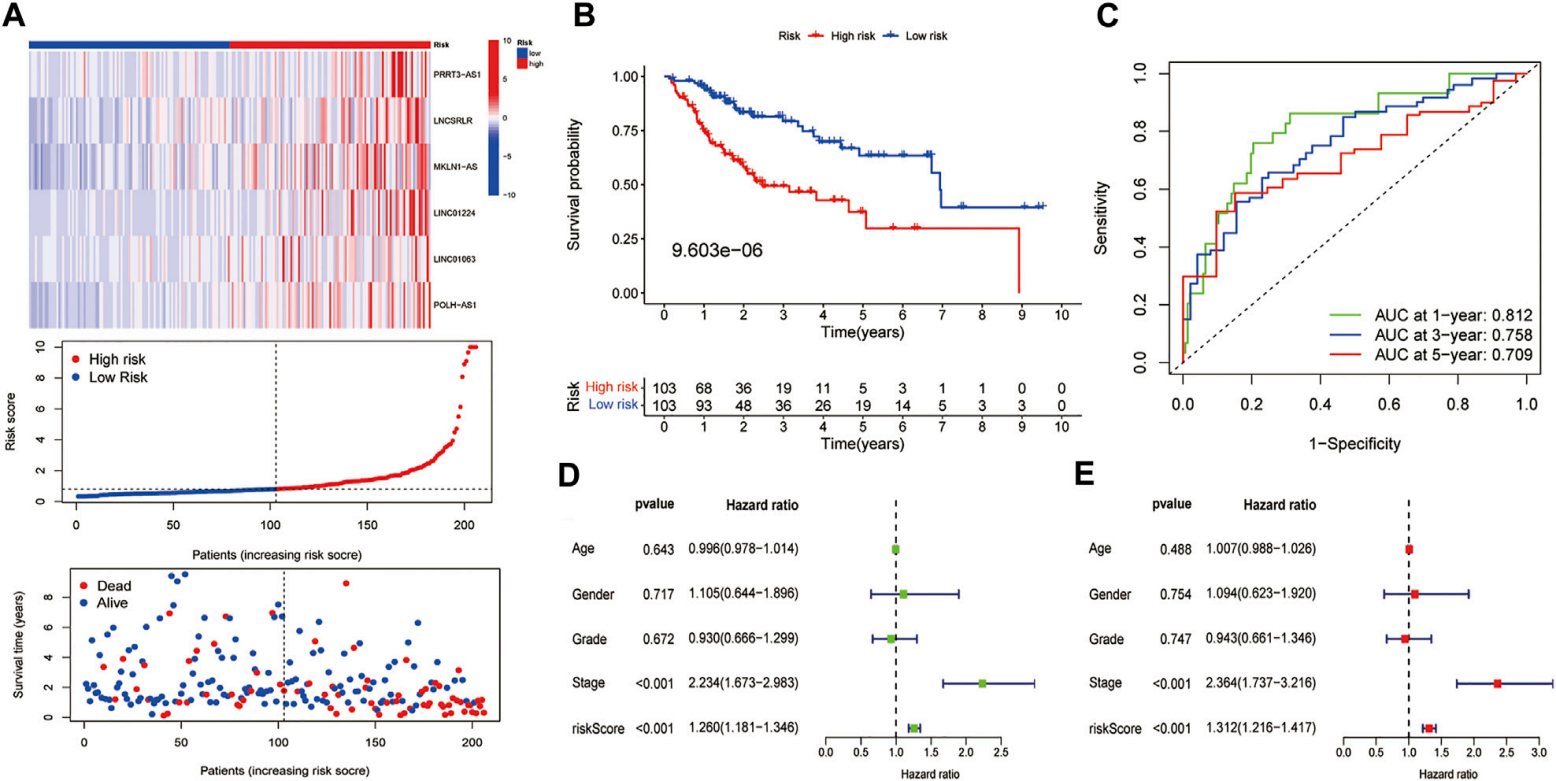
Preliminary examination of the model constructed from the training dataset. **(A)** The heatmap of the expression of six lncRNAs, risk scores, and survival status of each patient. **(B)** K–M survival analysis. **(C)** ROC analysis of the 1, 3, and 5 years OS of HCC patients. The **(D)** univariate and **(E)** multivariate Cox regression results in the training dataset.

We then calculated risk values for each case and categorised all cases as low and high risk based on the median threshold of the training dataset. As shown in the heat map, the expression of the six lncRNAs was higher in the high-risk group of HCC patients than in the low-risk group, gradually increasing patients’ mortality as risk score increased (Figure 3A). In the training dataset, K–M analysis showed that the survival rate of the low-risk group was significantly higher than that of the high-risk group (*p* = 9.603e-06) (Figure 3B). Then, we constructed an ROC curve and the results showed that the AUC values of 1, 3, and 5 years OS were 0.812, 0.758, and 0.709, respectively (Figure 3C). Furthermore, univariate and multivariate Cox regression analyses were performed on age, gender, tumour stage, histological grade, and risk score to verify whether the FR-related risk score could be used independently as an indicator of survival in OS. The results of univariate Cox regression analysis showed that tumour stage (*p* < 0.001) and risk score (*p* < 0.001) were associated with prognosis (Figure 3D). Multivariate Cox regression analysis illustrated that tumour stage (*p* < 0.001) or risk score (*p* < 0.001) could be used as an independent predictor of prognosis for HCC patients.

### Validation of the FR-lncRNA Signature

**FIGURE 4:**
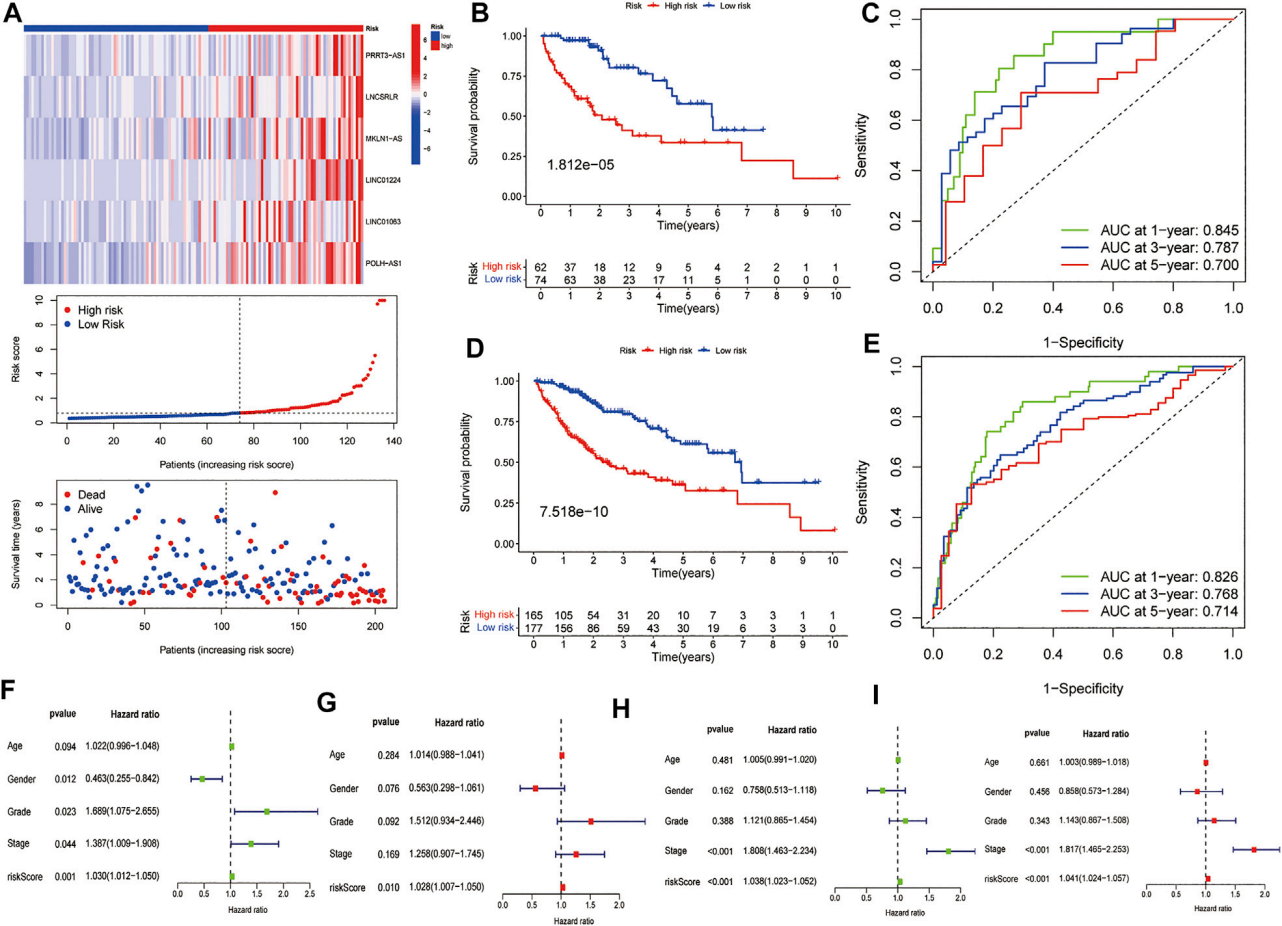
Further validation of the model in the testing group and the entire cohort. **(A)** The heatmap of the expression of six FR-lncRNAs, risk scores, and scattergram of every patient, **(B)** K–M curves for the OS and **(C)** ROC analysis of the 1, 3, and 5 years OS of HCC patients in the testing group. **(D)** K–M curves for the OS and **(E)** ROC curves of the 1, 3, and 5 years OS of HCC patients in the entire group. The **(F)** univariate and **(G)** multivariate Cox regression results in the testing group. The **(H)** univariate and **(I)** multivariate Cox regression results in the entire group.

**FIGURE 5:**
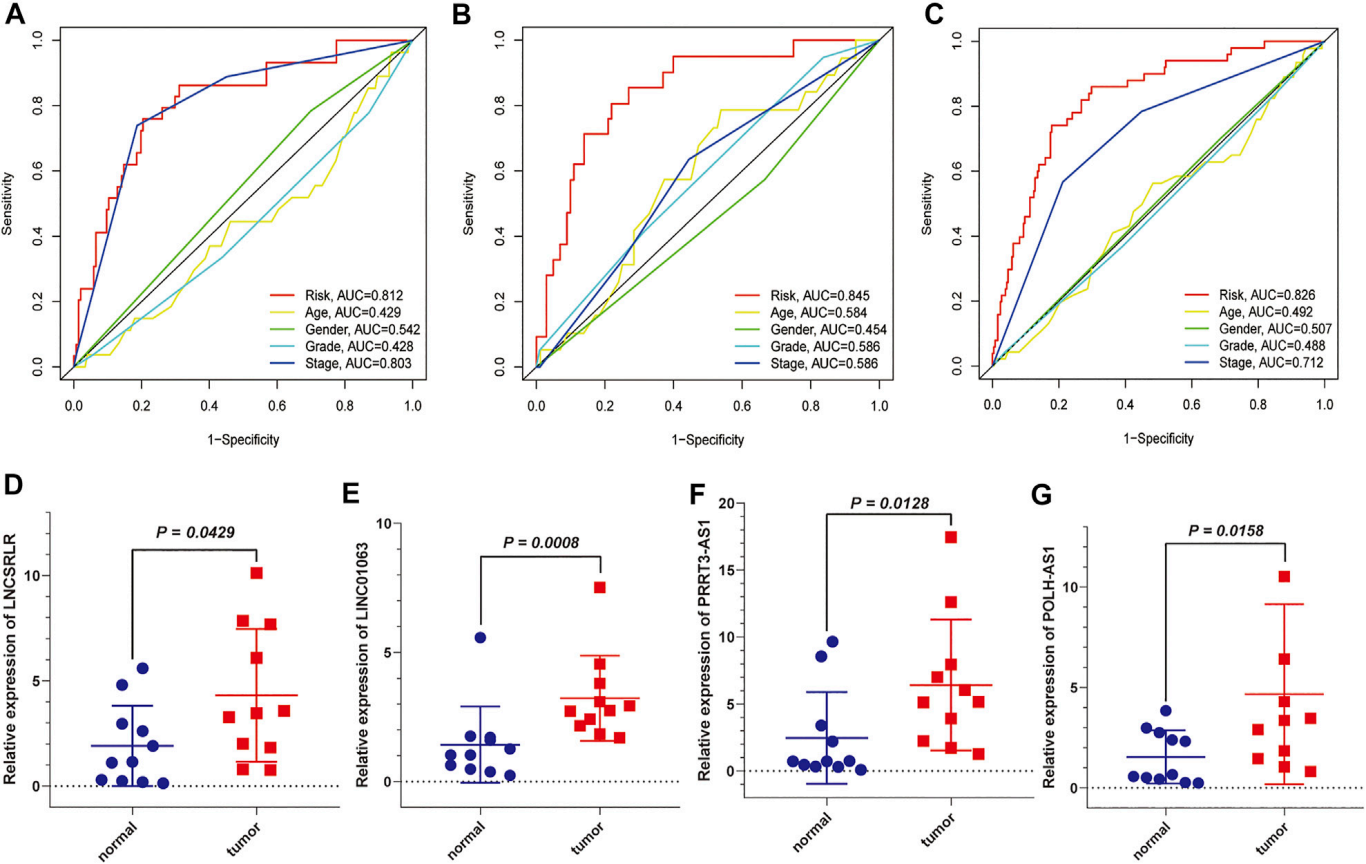
The AUC values of the six FR-lncRNAs model and clinical characteristics predicting 1-year survival and further validation by external experiments. **(A)** Training set. **(B)** Testing set. **(C)** Entire set. **(D)** LNCSRLR, **(E)** LINC01063, **(F)** POLH-AS1, and **(G)** PRRT3-AS1 are highly expressed in HCC tissues compared to normal tissues.

We used the test dataset and entire dataset to further validate the accuracy and reliability of the prognostic signature. In the test dataset, the distributions of expression profiles of the six-lncRNA, risk score, and OS status were consistent with those of the training dataset (Figure 4A). Similarly, K–M analysis of the testing set (Figure 4B) and entire data set (Figure 4D) showed that patients in the low-risk group had a higher survival rate than those in the high-risk group. The AUC values of the 1, 3, and 5 years OS in the test dataset were 0.845, 0.787, and 0.700, respectively (Figure 4C) and the AUC values of the 1, 3, and 5 years OS in the entire dataset were 0.826, 0.768, and 0.714, respectively (Figure 4E). Subsequently, we compared the accuracy of our signature with that of published articles according to the values of the AUC and concordance index (C-index) (Supplementary Figure S1). The efficacy of our signature is better than that of other studies using an identical protocol. Consistent with the results of the training group, therefore, the FR-lncRNA risk score could be used as an independent predictor of OS based on univariate and multivariate Cox regression analyses of the test dataset and the entire dataset (Figures 4F–I). Furthermore, to determine whether the risk score model could be used as the best predictor of survival, we included age, sex, tumour stage, and pathological grade as candidate predictive indicators. We analysed the AUC curves for 1-year prognosis in the three datasets and found that our signature possessed the highest AUC values among these factors (Figures 5A–C).

Significantly higher expression of MKLN1-AS and LINC01224 in HCC tissues compared to normal liver tissues has been demonstrated in previous studies (Dan Gong et al., 2020; Gao et al., 2020). We used qRT-PCR to detect the expression of the other four FR-lncRNAs in HCC tissues and adjacent normal tissues. The results showed that the expression levels of LNCSRLR (Figure 5D), LINC01063 (Figure 5E), PRRT3-AS1 (Figure 5F), and POLH-AS1 (Figure 5G) were higher in HCC tissues than in normal tissues.

### A Nomogram Combining the Risk Score, Age, and Tumour Stage to Predict Survival Time in Patients With HCC

**FIGURE 6:**
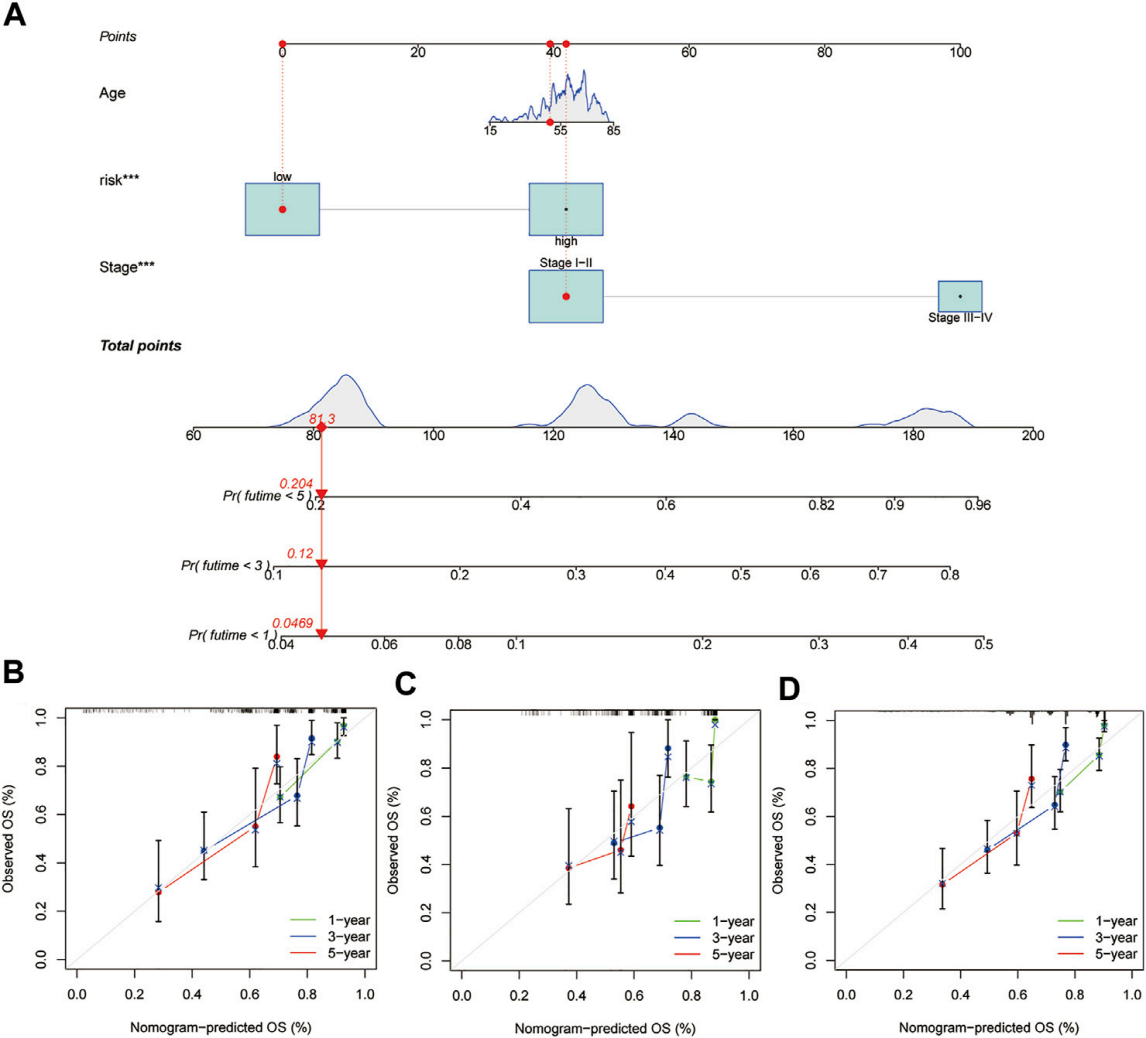
Construction of a nomogram in the training group and calibration maps were used to predict the 1-year, 3-years, and 5-years survival. **(A)** Nomogram constructed using the training group dataset. Assessing the accuracy of the nomogram using calibration maps in the **(B)** training group, **(C)** testing group, and **(D)** entire group.

Our prognostic nomogram integrated the six-lncRNA signature, age, and tumour stage. Then, we used the prognostic nomogram to predict the prognosis of HCC patients at one, three, and 5 years after diagnosis. (Figure 6A). Moreover, calibration plots of 1, 3 and 5 years survival probabilities also showed good agreement between nomogram predictions and actual observations (Figure 6B). Similar results have been observed for the testing set (Figure 6C) and the entire dataset (Figure 6D).

### Differences in Immune Cell Infiltration, Expression of Immune Checkpoint Genes and Sensitivity to Clinical Treatments Between High- and Low-Risk Groups

**FIGURE 7:**
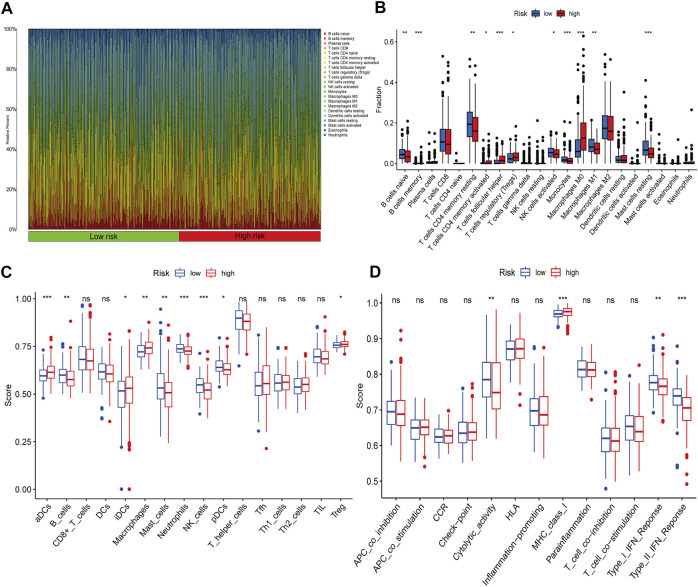
Immune profiles between different risk groups. **(A)** Relative proportions of immune cells in HCC patients. **(B)** Comparison of immune cell subtypes in the high- and low-risk groups. ssGSEA results show the differences in **(C)** immune cell infiltration and **(D)** immune function between the two groups.

The entire dataset was used to explore the relationship between the signature and immune cell infiltration, the expression of immune checkpoint genes, and clinical treatments. The differences in the infiltration of 22 immune cell types in the tumour tissue of all HCC patients are shown in Figure 7A. Patients with HCC in the high-risk group had higher ratios of M0 macrophages, follicular helper T cells, memory B cells, memory activated CD4 T cells, and regulatory T cells (Tregs) than those in the low-risk group (*p* < 0.05); meanwhile, patients with HCC in the low-risk group had higher ratios of activated NK cells, M1 macrophages, monocytes, naïve B cells, resting mast cells, and memory resting CD4 T cells than those in the high-risk group (*p* < 0.05) (Figure 7B). To explore the relationship between the expression of the six lnc-RNAs and immune infiltration level in HCC, lollipop plots were constructed showing Spearman’s correlation coefficient and statistical significance in Supplementary Figure S2. Notably, the six lnc-RNAs were positively correlated with the populations of M0 macrophages and Tregs and negatively correlated with the populations of monocytes and M2 macrophages (*p* < 0.05).

We also applied the ssGSEA method to the RNA sequencing data of HCC samples to assess immune cell infiltration and related functions. The populations of immune cells, including activated dendritic cells (aDCs), B cells, immature dendritic cells (iDCs), macrophages, mast cells, neutrophils, Natural Killer (NK) cells, plasmacytoid *dendritic* cells (pDCs), and Tregs, were found to be markedly different between the two groups (Figure 7C). Moreover, immune signature comparisons revealed that low-risk patients had higher cytolytic activity, type-I IFN response, and type-II IFN response than high-risk patients, whereas the opposite was observed for MHC class I (Figure 7D).

**FIGURE 8:**
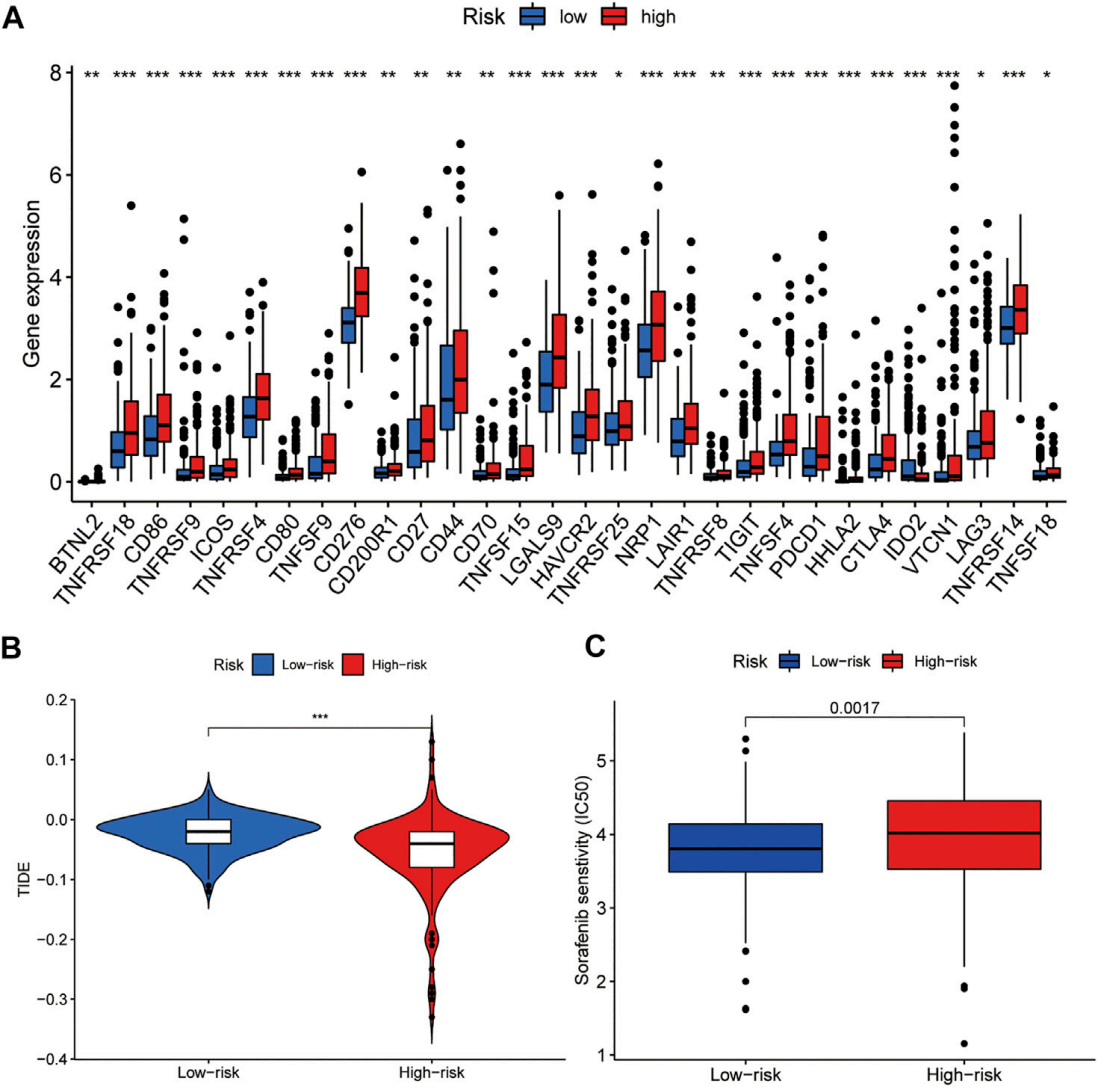
Comparison of the expression of immune checkpoint genes and sensitivity to clinical treatment between high- and low-risk groups. **(A)** Expression of immune checkpoint genes among the two groups. **(B)** TIDE score of the two groups. **(C)** The signature can be used as a potential predictor of sorafenib sensitivity, as low-risk scores are associated with a lower IC50 for sorafenib.

Given the importance of ICIs in the treatment of HCC, we further analysed the differential expression of immune checkpoint genes between the high- and low-risk groups. We found that patients in the high-risk group had higher expression of immune checkpoint genes, such as PDCD-1 (PD-1), CTLA4, LAG3, HAVCR2 (TIM3), and TIGIT (Figure 8A), compared to that in the low-risk group. We then used TIDE to assess the potential clinical efficacy of immunotherapy in the two groups. A higher TIDE prediction score represents a higher potential for immune evasion, suggesting that patients are less likely to benefit from ICI treatment. Our results indicated that the high-risk group had a lower TIDE score than that of the low-risk group, implying that patients in the high-risk group could benefit more from ICI therapy than patients in the low-risk group (Figure 8B). In addition to ICI treatment, we attempted to identify the association between the signature and the efficacy of sorafenib for the treatment of HCC. We found that the low-risk group was associated with lower IC50 for sorafenib (*p* < 0.01), which suggested that the signature could be used as a potential predictor of sorafenib treatment sensitivity (Figure 8C).

### Gene Mutation Analysis

**FIGURE 9:**
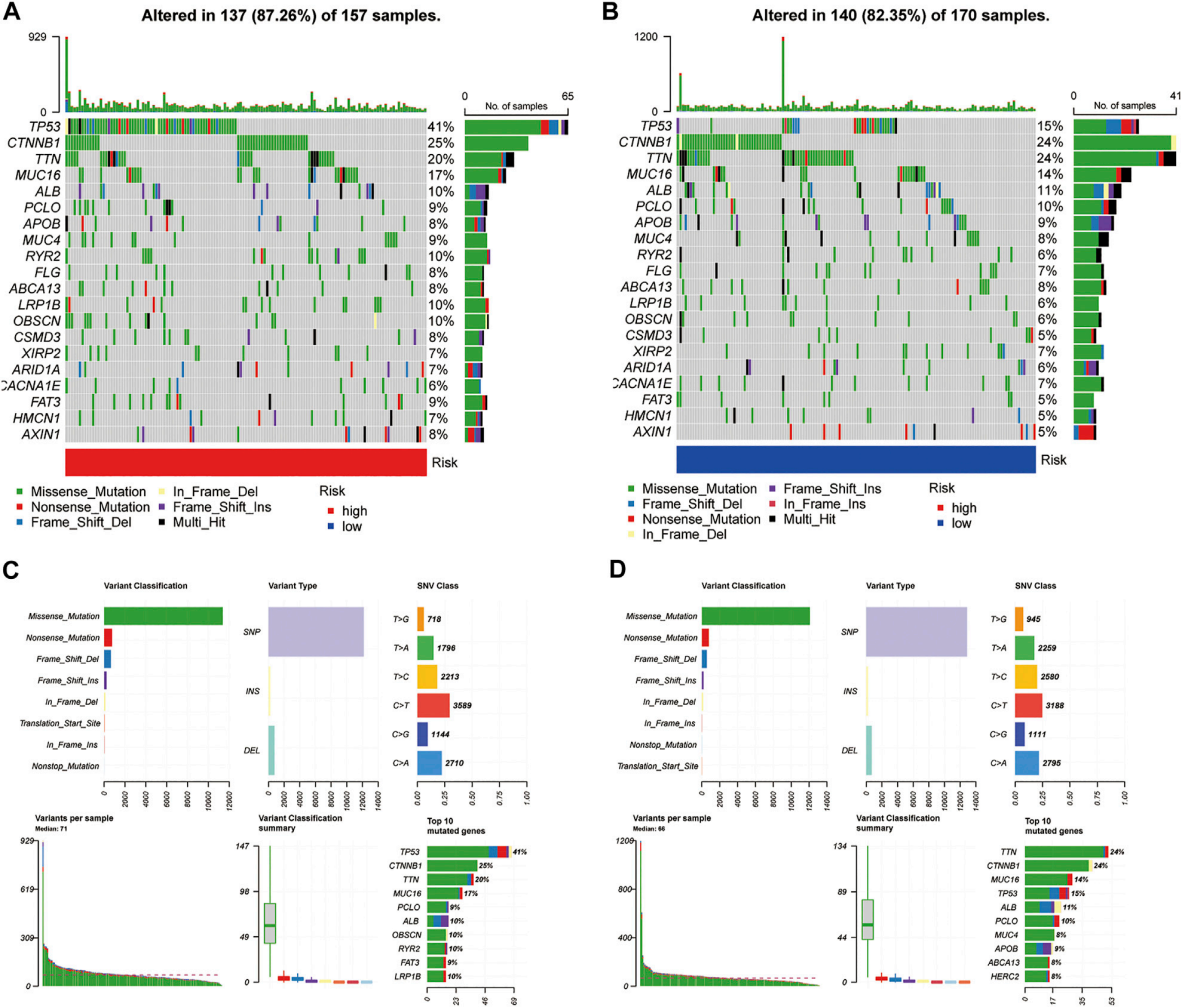
Somatic mutation analysis. **(A,B)** Oncoplots of the mutated genes in the **(A)** high-risk and **(B)** low-risk groups. [**(C,D)**, above] Missense mutations, SNPs, and C > T mutations were the most common mutation types in both groups. [**(C,D)**, below] Variants per sample, variant classification summary, and top ten mutated genes.

We processed simple nucleotide variation data using the “maftools” package in R. A waterfall plot displayed the top 20 mutated genes in patients with HCC (Figures 9A,B). A higher proportion of somatic mutations (TP53) were found in the high-risk group (FDR <0.01, *p <* 0.001) after a Fisher’s exact test was used to analyse the mutation differences between two groups. Figures 9C,D summarise the mutation information for the high- and low-risk gene groups, respectively.

### Construction of Co-Expression Network, CNV Analysis of FR Genes Associated With the Signature, and GSEA

**FIGURE 10:**
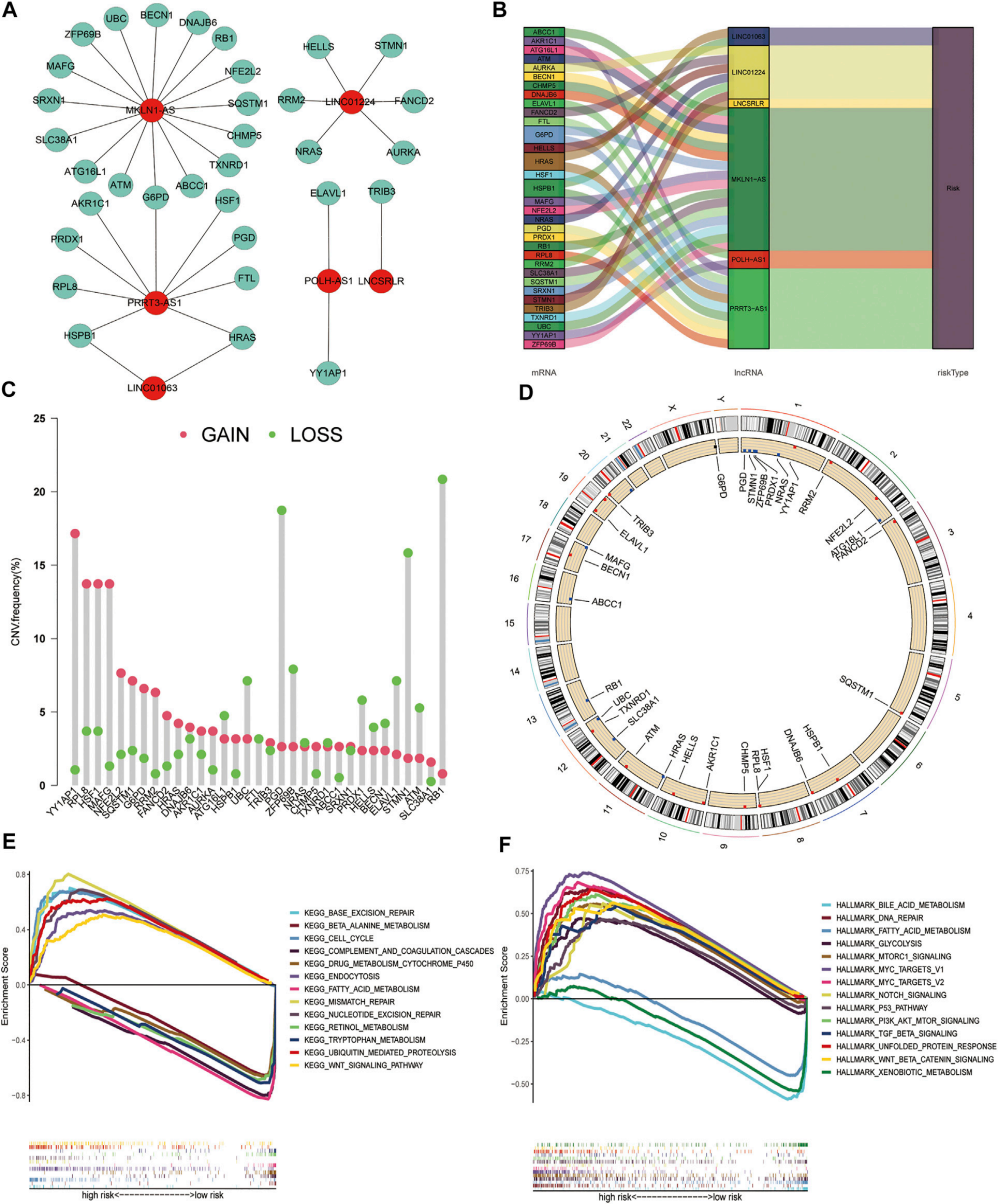
Construction of the co-expression network, copy number variations of ferroptosis genes associated with the signature, and Gene set enrichment analysis. **(A)** The Sankey plot shows the degree of association between the FR-lncRNAs and FRGs. **(B)** Diagram of the co-expression network. **(C)** The CNV frequency of FRGs associated with the signature. **(D)** The location of CNV changes of FRGs on chromosomes. **(E)** KEGG. **(F)** Hallmark gene set. (*p* < 0.05).

To explore the interaction between the six FR-lncRNAs and gene expression in HCC, Cytoscape was used to visualise the lncRNA and mRNA co-expression network (Figure 10A). The Sankey diagram showed the relationship between FR-lncRNAs, FRGs, and OS in patients with HCC (Figure 10B). It was found that the FRGs associated with signature had different levels of CNV events. RB1 harboured the most CNV events. Meanwhile, YY1AP1, RPL8, HSF1, MAFG, PGD, and STMN1 also had relatively high CNV events (Figure 10C). Figure 10D shows the location of CNV events in the FRGs related to the signature on the chromosome. Then, we performed GSEA to explore the biological effects of the six FR-lncRNAs signature, and the entire group dataset was used for GSEA analysis. The results revealed that the high-risk group showed significant enrichment in *Kyoto Encyclopedia of Genes and Genomes* (KEGG) pathways related to cancer processes, such as base excision repair, cell cycle, endocytosis, mismatch repair, nucleotide excision repair, and the WNT signal transduction pathway. Correspondingly, ferroptosis and metabolism-related pathways, such as beta-alanine metabolism, drug metabolism cytochrome P450, fatty acid metabolism, and retinol metabolism were significantly enriched in the low-risk group (Figure 10C). Furthermore, GSEA of the hallmark gene sets indicated that PI3K-AKT-MTOR, TGF-β, NOTCH, P53, and WNT-β-Catenin pathways were enriched in the high-risk group significantly, whereas fatty acid metabolism, bile acid metabolism, and xenobiotic metabolism pathways were highly enriched in the low-risk group (Figure 10D).

## Discussion

The prognosis for patients with HCC is poor, mainly because a large proportion of patients are diagnosed with HCC at an advanced stage (Llovet et al., 2021). Recently, despite the great success achieved using a combination of anti-PD-L1 with anti-VEGF therapies in advanced HCC, there are still a majority of patients who either do not respond to these treatments or do not have a lasting clinical benefit because of high tumour heterogeneity or treatment resistance(Finn et al., 2020; Pinter et al., 2020). Therefore, to maximize the benefits to patients and improve the effectiveness of systematic therapy, it is necessary to explore reliable molecular biomarkers to predict the effectiveness of immunotherapy, targeted therapies, and the prognosis of HCC patients.

LncRNAs play a key role in chromatin structure, cell growth, gene expression, differentiation, and development, and mutations or dysregulation of their expression are associated with a variety of diseases, particularly malignancies (Esteller, 2011; Bhan et al., 2017). Recent studies have revealed that lncRNAs are associated with the following six cancer hallmarks: proliferation, growth suppression, motility, immortality, angiogenesis, and viability (Schmitt and Chang, 2016). For the treatment of HCC, lncRNAs can be used as biomarkers to predict the efficacy of surgery, radiotherapy, chemotherapy, and immunotherapy, and are expected to be a potential tool for individualised HCC diagnosis and treatment (Yuan et al., 2021). Moreover, the TP53 mutation is involved in the expression of specific lnc-RNAs and gains oncogenic function by creating a complex network of interacting pathways (Di Agostino, 2020). For example, lincRNA-p21 serves as a transcriptional repressor in the p53 pathway and was the first lncRNA identified as being transcriptionally induced by wild-type p53 (Huarte et al., 2010). In 26 resected pancreatic cancer specimens, lncRNA1611 was significantly highly expressed in the cancerous pancreatic tissue of 22 patients compared to normal pancreatic tissue and was positively correlated with TP53 mutation (Wang et al., 2015). However, few studies have documented the relationship between TP53 mutation and lncRNA expression (Lin et al., 2019). Furthermore, several recent studies have found that lncRNAs are strongly associated with ferroptosis in cancer (Xie and Guo, 2021; Yao et al., 2021; Zhang et al., 2021; Zuli Wang et al., 2021). However, to date, the number of FR-associated lncRNAs identified in HCC is scarce, and research on FR-associated lncRNAs in HCC is limited.

RNA-targeted therapies are developing rapidly, such as the expression of HOTAIR-*sbid*, a mutant of lncRNA HOTAIR, which has been shown to reduce cell motility, invasiveness, and response to TGF β-induced epithelial-mesenchymal transition (Wang et al., 2020; Battistelli et al., 2021). In the era of precision medicine, by exploring FR-related lncRNAs, we aimed to obtain a risk model that could predict the efficacy of clinical treatments and patient prognosis. We successfully constructed and validated a new biomarker comprising six FR-related lncRNAs in patients with HCC based on TCGA dataset. Statistical analysis showed that this risk model has good robustness and predictive power and that it can independently predict OS in patients with HCC. Furthermore, the predictive accuracy of our signature surpassed that of the previously reported prediction signature of HCC dependent on FR-lncRNAs or FRGs (Liang et al., 2020; Chen et al., 2021; Liang Wang et al., 2021; Liang et al., 2021; Nie et al., 2021; Wan et al., 2021; Xu et al., 2021). The nomogram consisting of age, stage, and six FR-related lncRNA risk scores could be used to visually predict the OS of individual patients with HCC at 1, 3, and 5 years. According to the calibration plots, the nomogram has good prediction accuracy and the predicted results match the actual results well.

Sorafenib and immunotherapy can inhibit the progression of tumours, including HCC, by inducing ferroptosis (Louandre et al., 2013; Nie et al., 2018; Friedmann Angeli et al., 2019; Wang et al., 2019). Ferroptosis can promote exposure to tumour antigens, thereby enhancing the immunogenicity of the TIME and efficacy of immunotherapy (Zhang et al., 2019). Meanwhile, ICIs act primarily through the activation of an anti-tumour immune response driven by cytotoxic T cells, and this anti-tumour effect can induce ferroptosis in cancer cells (Chen et al., 2021a). Moreover, CD36 expressed by CD8^+^ T cells leads to an accumulation of lipid peroxides in CD8^+^ T cells through the uptake of fatty acids in the tumour environment, which in turn leads to an elevated iron ion content, increased iron death processes, and reduced secretion of cytotoxic cytokines (Ma et al., 2021). Therefore, HCC patients with different ferroptosis characteristics and immunophenotypes may respond differently to sorafenib or immunotherapy. In our study, the proportion of Tregs, M0 macrophages, follicular helper T cells, and memory B cell subsets in the CIBERSORT analysis showed significant infiltration in the high-risk group. The results of the ssGSEA analysis showed that the proportion of aDCs, iDCs, macrophages, and Tregs was significantly increased in the TIME of the high-risk group. In addition, immune checkpoint-associated genes were more highly expressed in patients of the high-risk group compared to the low-risk group, which may provide a basis for identifying patients who may respond to ICI therapy. In contrast, the infiltration of activated NK cells and M1 macrophages, which are immune-promoting cell subsets, was significantly increased in the low-risk group in the CIBERSORT analysis. Similarly, the results of the ssGSEA analysis showed that the low-risk group had a significantly higher proportion of B cells, neutrophils, and NK cells in the TIME, and significantly enhanced type-I IFN response, type-II IFN response, and cytolytic activity. Furthermore, the risk model could predict the sensitivity of HCC patients to sorafenib and ICI treatment. The results indicated that patients in the low-risk group were more sensitive to sorafenib than those in the high-risk group. Conversely, the high-risk group was more sensitive to immunotherapy. Importantly, these results may contribute to personalised immunotherapy and targeted therapy for patients with HCC.

Additionally, more patients in the high-risk group had TP53 somatic mutations than in the low-risk group (41% vs. 15%). TP53 mutations affect the cell cycle in approximately 30% of all HCC cases, and patients with this mutation tend to have a poor prognosis (Villanueva, 2019). Interestingly, the effect of TP53 mutations on ferroptosis showed different results depending on the mutation site (Jiang et al., 2015; Jennis et al., 2016). Furthermore, TP53 gene mutations can affect the recruitment and activity of bone marrow cells and T cells, leading to immune evasion and the promotion of cancer progression (Blagih et al., 2020). P53 functions in immune cells, leading to a variety of outcomes that can hinder or support tumour development (Blagih et al., 2020). Thus, a higher frequency of TP53 mutations in the high-risk group of HCC patients may be associated with the status of ferroptosis and an immunosuppressive phenotype. We also found that the FRGs related to our signature were all subject to varying degrees of CNVs, with the highest frequency of loss in the RB1 gene and the highest frequency of gains in YY1AP1. Although “RB1 loss of function” was present in <30% of HCC samples, both genes play an important role in HCC prognosis and treatment.

GSEA analysis results showed that the FR-related signalling pathways, such as P53 and TGF-β, were enriched in the high-risk group significantly, whereas fatty acid metabolism and drug metabolism cytochrome P450 signalling pathways were enriched in the low-risk group (Chen et al., 2021a; Chen et al., 2021b). Immune-related signalling pathways, such as mismatch repair, the Notch, P53, PI3K-AKT-MTOR, TGF-β, and WNT-β-Catenin pathways were significantly enriched in the high-risk group. These results provide further evidence for differences in ferroptosis and TIME characteristics between the two groups, which may serve as new therapeutic targets.

In the constructed FR-related lncRNA risk model, it was demonstrated that the expression of MKLN1-AS and LINC01224 was significantly upregulated in cell lines and HCC tissues and that the higher expression of MKLN1-AS and LINC01224 was associated with poorer prognosis (Dan Gong et al., 2020; Gao et al., 2020). In addition, MKLN1-AS, LINC01063, and PRRT3-AS1 can act as predictors of prognosis in patients with HCC through autophagy-related or immune-related pathways (Deng et al., 2020; Kong et al., 2020; Yang et al., 2021). However, the roles of MKLN1-AS, LINC01063, PRRT3-AS1, and LINC01224 in the ferroptosis pathway have not yet been reported. LNCSRLR can be used as a biomarker of prognosis in patients with laryngeal squamous cell carcinoma; however, its role in HCC is unknown (Shiqi Gong et al., 2020). The function or pathway of POLH-AS1 has not yet been reported. Furthermore, the expression levels of LNCSRLR, LINC01063, PRRT3-AS1, and POLH-AS1 in HCC tissues have not been determined. We demonstrated that the expression of LNCSRLR, LINC01063, PRRT3-AS1, and POLH-AS1 was higher in HCC tissues than in normal tissues using qPCR.

Our study has certain limitations. First, further basic experiments are needed to validate the relationship between the lncRNAs screened using co-expression analysis and ferroptosis. Our study provides the basis for further in-depth research. Second, we were unable to retrieve a dataset that simultaneously reported six-lncRNA expression levels, clinical characteristics, and survival status of HCC patients. Therefore, we did not perform an external validation of the signature.

In conclusion, we constructed a novel FR-lncRNA signature with favourable specificity and sensitivity for predicting survival time in patients with HCC. A nomogram constructed using age, clinical TNM staging, and risk scores for the six FR-lncRNAs can be a simple tool to predict the survival time of HCC patients. More importantly, our signature can also predict the efficacy of immunotherapy and targeted therapies, which is important for reducing patient suffering, improving the effectiveness of drug treatment, and saving healthcare resources.

## Data Availability

The datasets presented in this study can be found in online repositories. The names of the repository/repositories and accession number(s) can be found in the article/Supplementary Material.

## References

[B1] Battistelli C. Garbo S. Riccioni V. Montaldo C. Santangelo L. Vandelli A. (2021). Design and Functional Validation of a Mutant Variant of the LncRNA HOTAIR to Counteract Snail Function in Epithelial-To-Mesenchymal Transition. Cancer Res. 81 (1), 103–113. 10.1158/0008-5472.Can-20-1764 33158813 PMC7611326

[B2] Becht E. Giraldo N. A. Lacroix L. Buttard B. Elarouci N. Petitprez F. (2016). Estimating the Population Abundance of Tissue-Infiltrating Immune and Stromal Cell Populations Using Gene Expression. Genome Biol. 17 (1), 218. 10.1186/s13059-016-1070-5 27765066 PMC5073889

[B3] Bhan A. Soleimani M. Mandal S. S. (2017). Long Noncoding RNA and Cancer: A New Paradigm. Cancer Res. 77 (15), 3965–3981. 10.1158/0008-5472.Can-16-2634 28701486 PMC8330958

[B4] Blagih J. Buck M. D. Vousden K. H. (2020). p53, Cancer and the Immune Response. J. Cel Sci 133 (5), jcs237453. 10.1242/jcs.237453 32144194

[B5] Capelletti M. M. Manceau H. Puy H. Peoc’h K. (2020). Ferroptosis in Liver Diseases: An Overview. Int. J. Mol. Sci. 21 (14), 4908. 10.3390/ijms21144908 PMC7404091 32664576

[B6] Chen X. Kang R. Kroemer G. Tang D. (2021a). Broadening Horizons: the Role of Ferroptosis in Cancer. Nat. Rev. Clin. Oncol. 18 (5), 280–296. 10.1038/s41571-020-00462-0 33514910

[B7] Chen X. Yu C. Kang R. Kroemer G. Tang D. (2021b). Cellular Degradation Systems in Ferroptosis. Cell Death Differ 28 (4), 1135–1148. 10.1038/s41418-020-00728-1 33462411 PMC8027807

[B8] Chen Z.-A. Tian H. Yao D.-M. Zhang Y. Feng Z.-J. Yang C.-J. (2021). Identification of a Ferroptosis-Related Signature Model Including mRNAs and lncRNAs for Predicting Prognosis and Immune Activity in Hepatocellular Carcinoma. Front. Oncol. 11, 738477. 10.3389/fonc.2021.738477 34568075 PMC8458836

[B9] Deng X. Bi Q. Chen S. Chen X. Li S. Zhong Z. (2020). Identification of a Five-Autophagy-Related-lncRNA Signature as a Novel Prognostic Biomarker for Hepatocellular Carcinoma. Front. Mol. Biosci. 7, 611626. 10.3389/fmolb.2020.611626 33505990 PMC7831610

[B10] Di Agostino S. (2020). The Impact of Mutant P53 in the Non-coding RNA World. Biomolecules 10 (3), 472. 10.3390/biom10030472 PMC7175150 32204575

[B11] Esteller M. (2011). Non-coding RNAs in Human Disease. Nat. Rev. Genet. 12 (12), 861–874. 10.1038/nrg3074 22094949

[B12] Finn R. S. Qin S. Ikeda M. Galle P. R. Ducreux M. Kim T.-Y. (2020). Atezolizumab Plus Bevacizumab in Unresectable Hepatocellular Carcinoma. N. Engl. J. Med. 382 (20), 1894–1905. 10.1056/NEJMoa1915745 32402160

[B13] Friedmann Angeli J. P. Krysko D. V. Conrad M. (2019). Ferroptosis at the Crossroads of Cancer-Acquired Drug Resistance and Immune Evasion. Nat. Rev. Cancer 19 (7), 405–414. 10.1038/s41568-019-0149-1 31101865

[B14] Gao W. Chen X. Chi W. Xue M. (2020). Long Non‑coding RNA MKLN1‑AS Aggravates Hepatocellular Carcinoma Progression by Functioning as a Molecular Sponge for miR‑654‑3p, Thereby Promoting Hepatoma‑derived Growth Factor Expression. Int. J. Mol. Med. 46 (5), 1743–1754. 10.3892/ijmm.2020.4722 33000222 PMC7521589

[B15] Gong D. Feng P.-C. Ke X.-F. Kuang H.-L. Pan L.-L. Ye Q. (2020). Silencing Long Non-coding RNA LINC01224 Inhibits Hepatocellular Carcinoma Progression via MicroRNA-330-5p-Induced Inhibition of CHEK1. Mol. Ther. - Nucleic Acids 19, 482–497. 10.1016/j.omtn.2019.10.007 31902747 PMC6948252

[B16] Gong S. Xu M. Zhang Y. Shan Y. Zhang H. (2020). The Prognostic Signature and Potential Target Genes of Six Long Non-coding RNA in Laryngeal Squamous Cell Carcinoma. Front. Genet. 11, 413. 10.3389/fgene.2020.00413 32411183 PMC7198905

[B17] Huarte M. Guttman M. Feldser D. Garber M. Koziol M. J. Kenzelmann-Broz D. (2010). A Large Intergenic Noncoding RNA Induced by P53 Mediates Global Gene Repression in the P53 Response. Cell 142 (3), 409–419. 10.1016/j.cell.2010.06.040 20673990 PMC2956184

[B18] Jennis M. Kung C.-P. Basu S. Budina-Kolomets A. Leu J. I.-J. Khaku S. (2016). An African-specific Polymorphism in the TP53 Gene Impairs P53 Tumor Suppressor Function in a Mouse Model. Genes Dev. 30 (8), 918–930. 10.1101/gad.275891.115 27034505 PMC4840298

[B19] Jiang L. Kon N. Li T. Wang S.-J. Su T. Hibshoosh H. (2015). Ferroptosis as a P53-Mediated Activity during Tumour Suppression. Nature 520 (7545), 57–62. 10.1038/nature14344 25799988 PMC4455927

[B20] Jiang P. Gu S. Pan D. Fu J. Sahu A. Hu X. (2018). Signatures of T Cell Dysfunction and Exclusion Predict Cancer Immunotherapy Response. Nat. Med. 24 (10), 1550–1558. 10.1038/s41591-018-0136-1 30127393 PMC6487502

[B21] Kanehisa M. Furumichi M. Tanabe M. Sato Y. Morishima K. (2017). KEGG: New Perspectives on Genomes, Pathways, Diseases and Drugs. Nucleic Acids Res. 45 (D1), D353–d361. 10.1093/nar/gkw1092 27899662 PMC5210567

[B22] Kong W. Wang X. Zuo X. Mao Z. Cheng Y. Chen W. (2020). Development and Validation of an Immune-Related lncRNA Signature for Predicting the Prognosis of Hepatocellular Carcinoma. Front. Genet. 11, 1037. 10.3389/fgene.2020.01037 33101369 PMC7500314

[B23] Li D. Liu X. Zhou J. Hu J. Zhang D. Liu J. (2017). Long Noncoding RNA HULC Modulates the Phosphorylation of YB-1 through Serving as a Scaffold of Extracellular Signal-Regulated Kinase and YB-1 to Enhance Hepatocarcinogenesis. Hepatology 65 (5), 1612–1627. 10.1002/hep.29010 28027578

[B24] Liang J.-y. Wang D.-s. Lin H.-c. Chen X.-x. Yang H. Zheng Y. (2020). A Novel Ferroptosis-Related Gene Signature for Overall Survival Prediction in Patients with Hepatocellular Carcinoma. Int. J. Biol. Sci. 16 (13), 2430–2441. 10.7150/ijbs.45050 32760210 PMC7378635

[B25] Liang J. Zhi Y. Deng W. Zhou W. Li X. Cai Z. (2021). Development and Validation of Ferroptosis-Related lncRNAs Signature for Hepatocellular Carcinoma. PeerJ 9, e11627. 10.7717/peerj.11627 34178478 PMC8202323

[B26] Lin T. Hou P.-F. Meng S. Chen F. Jiang T. Li M.-L. (2019). Emerging Roles of P53 Related lncRNAs in Cancer Progression: A Systematic Review. Int. J. Biol. Sci. 15 (6), 1287–1298. 10.7150/ijbs.33218 31223287 PMC6567798

[B27] Llovet J. M. Kelley R. K. Villanueva A. Singal A. G. Pikarsky E. Roayaie S. (2021). Hepatocellular Carcinoma. Nat. Rev. Dis. Primers 7 (1), 6. 10.1038/s41572-020-00240-3 33479224 PMC13537433

[B28] Louandre C. Ezzoukhry Z. Godin C. Barbare J.-C. Mazière J.-C. Chauffert B. (2013). Iron-dependent Cell Death of Hepatocellular Carcinoma Cells Exposed to Sorafenib. Int. J. Cancer 133 (7), 1732–1742. 10.1002/ijc.28159 23505071

[B29] Ma X. Xiao L. Liu L. Ye L. Su P. Bi E. (2021). CD36-mediated Ferroptosis Dampens Intratumoral CD8+ T Cell Effector Function and Impairs Their Antitumor Ability. Cel Metab. 33 (5), 1001–1012.e5. 10.1016/j.cmet.2021.02.015 PMC8102368 33691090

[B30] Mai H. Zhou B. Liu L. Yang F. Conran C. Ji Y. (2019). Molecular Pattern of lncRNAs in Hepatocellular Carcinoma. J. Exp. Clin. Cancer Res. 38 (1), 198. 10.1186/s13046-019-1213-0 31097003 PMC6524221

[B31] Newman A. M. Liu C. L. Green M. R. Gentles A. J. Feng W. Xu Y. (2015). Robust Enumeration of Cell Subsets from Tissue Expression Profiles. Nat. Methods 12 (5), 453–457. 10.1038/nmeth.3337 25822800 PMC4739640

[B32] Newman A. M. Steen C. B. Liu C. L. Gentles A. J. Chaudhuri A. A. Scherer F. (2019). Determining Cell Type Abundance and Expression from Bulk Tissues with Digital Cytometry. Nat. Biotechnol. 37 (7), 773–782. 10.1038/s41587-019-0114-2 31061481 PMC6610714

[B33] Nie J. Lin B. Zhou M. Wu L. Zheng T. (2018). Role of Ferroptosis in Hepatocellular Carcinoma. J. Cancer Res. Clin. Oncol. 144 (12), 2329–2337. 10.1007/s00432-018-2740-3 30167889 PMC11813439

[B34] Nie Y. Li J. Wu W. Guo D. Lei X. Zhang T. (2021). A Novel Nine-lncRNA Risk Signature Correlates with Immunotherapy in Hepatocellular Carcinoma. Front. Oncol. 11, 706915. 10.3389/fonc.2021.706915 34604045 PMC8479152

[B35] Pinter M. Jain R. K. Duda D. G. (2021). The Current Landscape of Immune Checkpoint Blockade in Hepatocellular Carcinoma. JAMA Oncol. 7, 113. 10.1001/jamaoncol.2020.3381 33090190 PMC8265820

[B36] Qi W. Li Z. Xia L. Dai J. Zhang Q. Wu C. (2019). LncRNA GABPB1-AS1 and GABPB1 Regulate Oxidative Stress during Erastin-Induced Ferroptosis in HepG2 Hepatocellular Carcinoma Cells. Sci. Rep. 9 (1), 16185. 10.1038/s41598-019-52837-8 31700067 PMC6838315

[B37] Quinn J. J. Chang H. Y. (2016). Unique Features of Long Non-coding RNA Biogenesis and Function. Nat. Rev. Genet. 17 (1), 47–62. 10.1038/nrg.2015.10 26666209

[B38] Schmitt A. M. Chang H. Y. (2016). Long Noncoding RNAs in Cancer Pathways. Cancer Cell 29 (4), 452–463. 10.1016/j.ccell.2016.03.010 27070700 PMC4831138

[B39] Stockwell B. R. Friedmann Angeli J. P. Bayir H. Bush A. I. Conrad M. Dixon S. J. (2017). Ferroptosis: A Regulated Cell Death Nexus Linking Metabolism, Redox Biology, and Disease. Cell 171 (2), 273–285. 10.1016/j.cell.2017.09.021 28985560 PMC5685180

[B40] Subramanian A. Tamayo P. Mootha V. K. Mukherjee S. Ebert B. L. Gillette M. A. (2005). Gene Set Enrichment Analysis: a Knowledge-Based Approach for Interpreting Genome-wide Expression Profiles. Proc. Natl. Acad. Sci. 102 (43), 15545–15550. 10.1073/pnas.0506580102 16199517 PMC1239896

[B41] Villanueva A. (2019). Hepatocellular Carcinoma. N. Engl. J. Med. 380 (15), 1450–1462. 10.1056/NEJMra1713263 30970190

[B42] Wan S. Lei Y. Li M. Wu B. (2021). A Prognostic Model for Hepatocellular Carcinoma Patients Based on Signature Ferroptosis-Related Genes. Hepatol. Int. [Epub ahead of print] 10.1007/s12072-021-10248-w 34449009

[B43] Wang F. Zuroske T. Watts J. K. (2020). RNA Therapeutics on the Rise. Nat. Rev. Drug Discov. 19 (7), 441–442. 10.1038/d41573-020-00078-0 32341501

[B44] Wang L. Ge X. Zhang Z. Ye Y. Zhou Z. Li M. (2021). Identification of a Ferroptosis-Related Long Noncoding RNA Prognostic Signature and its Predictive Ability to Immunotherapy in Hepatocellular Carcinoma. Front. Genet. 12, 682082. 10.3389/fgene.2021.682082 34745200 PMC8566703

[B45] Wang Q. Jiang H. Ping C. Shen R. Liu T. Li J. (2015). Exploring the Wnt Pathway-Associated LncRNAs and Genes Involved in Pancreatic Carcinogenesis Driven by Tp53 Mutation. Pharm. Res. 32 (3), 793–805. 10.1007/s11095-013-1269-z 24469904

[B46] Wang W. Green M. Choi J. E. Gijón M. Kennedy P. D. Johnson J. K. (2019). CD8+ T Cells Regulate Tumour Ferroptosis during Cancer Immunotherapy. Nature 569 (7755), 270–274. 10.1038/s41586-019-1170-y 31043744 PMC6533917

[B47] Wang Z. Chen X. Liu N. Shi Y. Liu Y. Ouyang L. (2021). A Nuclear Long Non-coding RNA LINC00618 Accelerates Ferroptosis in a Manner Dependent upon Apoptosis. Mol. Ther. 29 (1), 263–274. 10.1016/j.ymthe.2020.09.024 33002417 PMC7791008

[B48] Xie B. Guo Y. (2021). Molecular Mechanism of Cell Ferroptosis and Research Progress in Regulation of Ferroptosis by Noncoding RNAs in Tumor Cells. Cell Death Discov. 7 (1), 101. 10.1038/s41420-021-00483-3 33980834 PMC8115351

[B49] Xu Z. Peng B. Liang Q. Chen X. Cai Y. Zeng S. (2021). Construction of a Ferroptosis-Related Nine-lncRNA Signature for Predicting Prognosis and Immune Response in Hepatocellular Carcinoma. Front. Immunol. 12, 719175. 10.3389/fimmu.2021.719175 34603293 PMC8484522

[B50] Yan X. Zhang D. Wu W. Wu S. Qian J. Hao Y. (2017). Mesenchymal Stem Cells Promote Hepatocarcinogenesis via lncRNA-MUF Interaction with ANXA2 and miR-34a. Cancer Res. 77 (23), 6704–6716. 10.1158/0008-5472.Can-17-1915 28947421

[B51] Yang S. Zhou Y. Zhang X. Wang L. Fu J. Zhao X. (2021). The Prognostic Value of an Autophagy-Related lncRNA Signature in Hepatocellular Carcinoma. BMC Bioinformatics 22 (1), 217. 10.1186/s12859-021-04123-6 33910497 PMC8080392

[B52] Yao J. Chen X. Liu X. Li R. Zhou X. Qu Y. (2021). Characterization of a Ferroptosis and Iron-Metabolism Related lncRNA Signature in Lung Adenocarcinoma. Cancer Cel Int 21 (1), 340. 10.1186/s12935-021-02027-2 PMC8254945 34217273

[B53] Yuan D. Chen Y. Li X. Li J. Zhao Y. Shen J. (2021). Long Non-coding RNAs: Potential Biomarkers and Targets for Hepatocellular Carcinoma Therapy and Diagnosis. Int. J. Biol. Sci. 17 (1), 220–235. 10.7150/ijbs.50730 33390845 PMC7757045

[B54] Zhang F. Li F. Lu G.-H. Nie W. Zhang L. Lv Y. (2019). Engineering Magnetosomes for Ferroptosis/Immunomodulation Synergism in Cancer. ACS Nano 13 (5), 5662–5673. 10.1021/acsnano.9b00892 31046234

[B55] Zhang J. Li Z. Liu L. Wang Q. Li S. Chen D. (2018). Long Noncoding RNA TSLNC8 Is a Tumor Suppressor that Inactivates the interleukin-6/STAT3 Signaling Pathway. Hepatology 67 (1), 171–187. 10.1002/hep.29405 28746790

[B56] Zhang Y. Guo S. Wang S. Li X. Hou D. Li H. (2021). LncRNA OIP5-AS1 Inhibits Ferroptosis in Prostate Cancer with Long-Term Cadmium Exposure through miR-128-3p/SLC7A11 Signaling. Ecotoxicology Environ. Saf. 220, 112376. 10.1016/j.ecoenv.2021.112376 34051661

[B57] Zhou N. Bao J. (2020). FerrDb: a Manually Curated Resource for Regulators and Markers of Ferroptosis and Ferroptosis-Disease Associations. Database (Oxford) 2020, baaa021. 10.1093/database/baaa021 32219413 PMC7100629

