# Supplementary figures and images for: Construction and Validation of a Ferroptosis-Related lncRNA Signature as a Novel Biomarker for Prognosis, Immunotherapy and Targeted Therapy in Hepatocellular Carcinoma
Source: Front Cell Dev Biol. 2022 Feb 22;10:792676. doi: 10.3389/fcell.2022.792676 (PMC8919262; doi:10.3389/fcell.2022.792676)

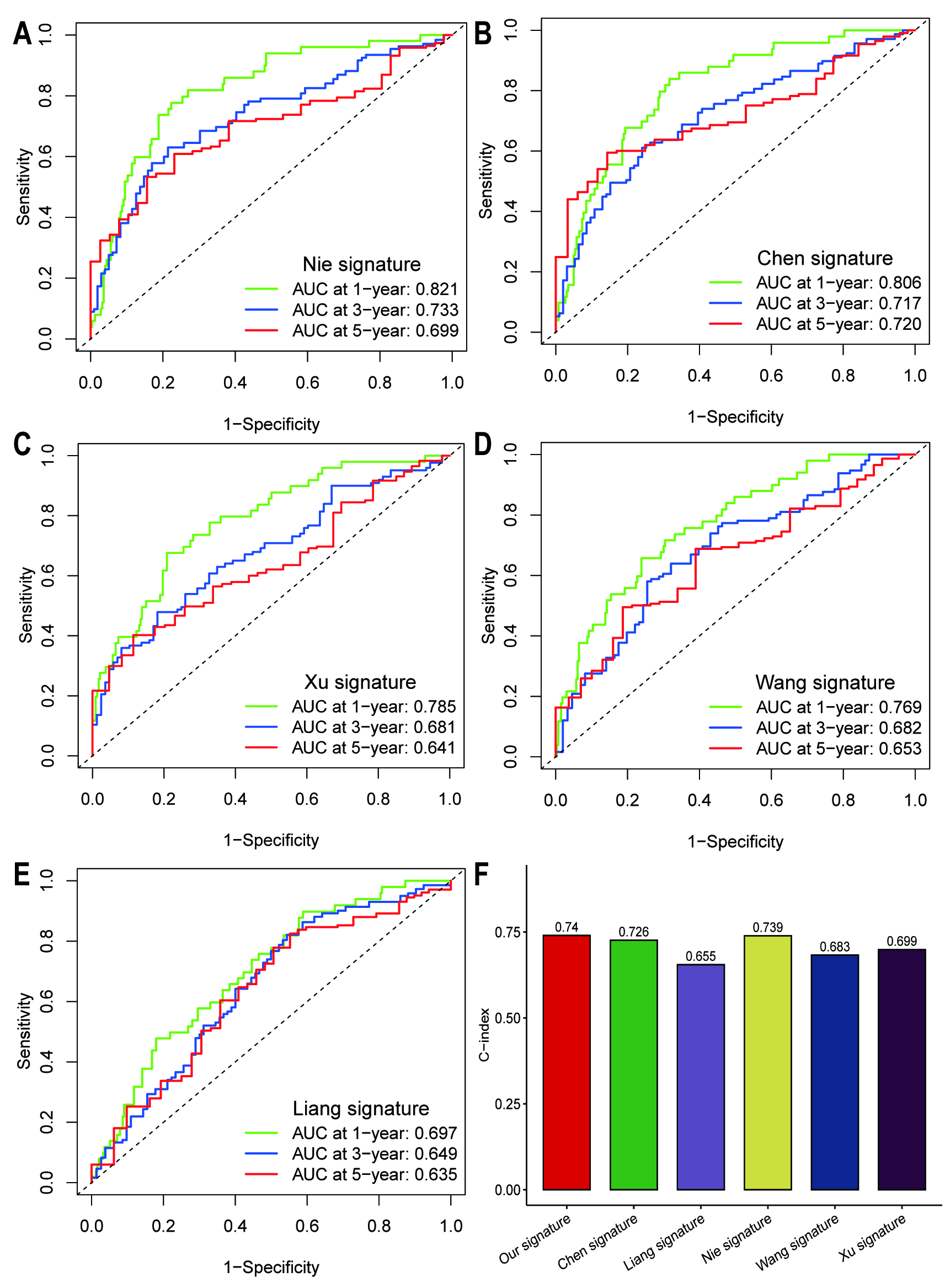

Supplement: Supplementary file 1 [file Image11.tif]

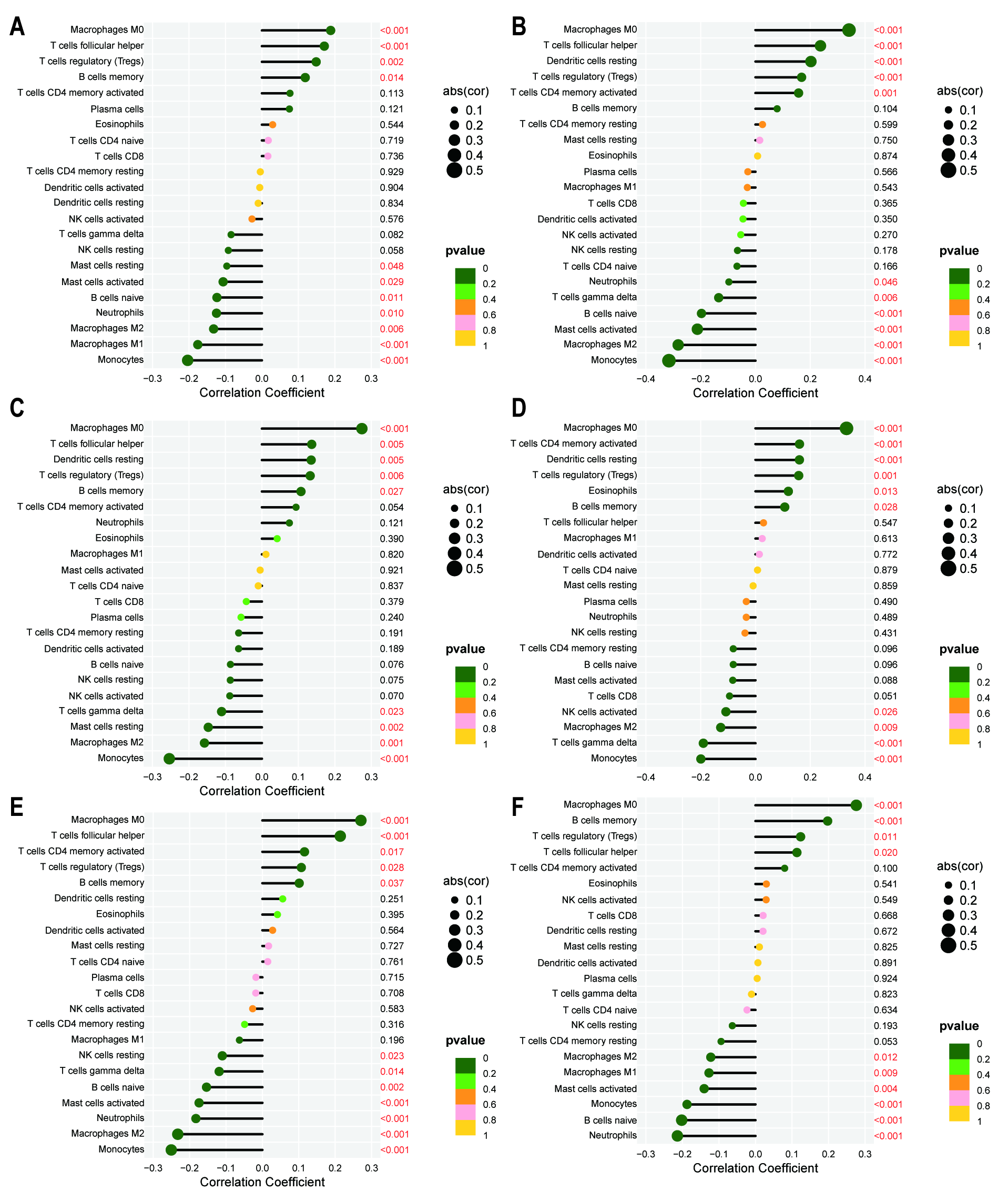

Supplement: Supplementary file 5 [file Image12.tif]
