# Supplementary material for: Construction and Validation of a Ferroptosis-Related lncRNA Signature as a Novel Biomarker for Prognosis, Immunotherapy and Targeted Therapy in Hepatocellular Carcinoma
Source: Front Cell Dev Biol. 2022 Feb 22;10:792676. doi: 10.3389/fcell.2022.792676 (PMC8919262; doi:10.3389/fcell.2022.792676)
Supplement: Supplementary file 2 [file Table2.DOCX]

Table S1. All the primer sequences of the article.

**PRRT3-AS1** forward: 5’-TCAACAACGCCCCTCTGAAAG-3’

reverse: 5’- AGTGTGAGGCCGTATGAATGG-3’;

**LNCSRLR** forward: 5’- TGCCAACTCATCGGAACAGAC-3’

reverse: 5’- ATTAAGCTGGCTTGCACTGGTA-3’;

**LINC01063** forward: 5’-TGCCGAGTGTGGTTTGCTATC-3’

reverse: 5’- GCCAATCACCTTCCAGGCTCAG -3’;

**POLH-AS1** forward: 5’-CTGCAGCCTCTAGCTTGACAT-3’

reverse:5’- CTGCCCAGGGAAGCTTGTGA-3’;

**β-actin** forward: 5’-TGACGTGGACATCCGCAAAG-3’

reverse: 5’-CTGGAAGGTGGACAGCGAGG-3’.
