# Supplementary material for: Construction and Validation of a Ferroptosis-Related lncRNA Signature as a Novel Biomarker for Prognosis, Immunotherapy and Targeted Therapy in Hepatocellular Carcinoma
Source: Front Cell Dev Biol. 2022 Feb 22;10:792676. doi: 10.3389/fcell.2022.792676 (PMC8919262; doi:10.3389/fcell.2022.792676)
Supplement: Supplementary file 3 [file Table3.DOCX]

| gene | HR | HR.95L | HR.95H | pvalue |
| --- | --- | --- | --- | --- |
| PVT1 | 1.129333044 | 1.001530377 | 1.273444275 | 0.047153352 |
| WARS2-AS1 | 1.384058533 | 1.017249077 | 1.883135671 | 0.038562809 |
| SUV39H2-DT | 1.604314966 | 1.197149912 | 2.149961742 | 0.001552561 |
| STK24-AS1 | 1.923129299 | 1.028308621 | 3.596611197 | 0.040622783 |
| LINC00623 | 1.258436055 | 1.087219464 | 1.456616036 | 0.002065355 |
| BSG-AS1 | 1.177870849 | 1.083828419 | 1.280073223 | 0.000115204 |
| SNHG7 | 1.032731471 | 1.007928495 | 1.058144796 | 0.009413257 |
| ZNF8-DT | 1.389316743 | 1.131247705 | 1.7062585 | 0.001711544 |
| THUMPD3-AS1 | 1.417562012 | 1.208169543 | 1.663245087 | 1.88E-05 |
| MIR4435-2HG | 1.129790359 | 1.049061664 | 1.216731389 | 0.001254397 |
| NRSN2-AS1 | 1.291849383 | 1.09397134 | 1.525519697 | 0.002538244 |
| CYTOR | 1.036757121 | 1.01324841 | 1.060811266 | 0.002037978 |
| CTC-338M12.4 | 2.061673544 | 1.090463895 | 3.897880362 | 0.025982978 |
| LINC01138 | 1.433682592 | 1.232377975 | 1.667869611 | 3.06E-06 |
| ARRDC1-AS1 | 1.109625509 | 1.027721842 | 1.198056439 | 0.00783923 |
| BACE1-AS | 1.226284422 | 1.1093868 | 1.355499708 | 6.58E-05 |
| MAPKAPK5-AS1 | 1.071114217 | 1.013055762 | 1.132500015 | 0.015684969 |
| STARD7-AS1 | 1.448668155 | 1.002626095 | 2.093142631 | 0.048389547 |
| ZNF433-AS1 | 1.935441507 | 1.188431969 | 3.151996855 | 0.007960375 |
| TMCC1-DT | 2.776262134 | 1.933730474 | 3.985887146 | 3.13E-08 |
| HNRNPD-DT | 2.407333879 | 1.268186259 | 4.569720232 | 0.007220389 |
| RUSC1-AS1 | 1.121147292 | 1.006606674 | 1.248721356 | 0.037550848 |
| SCAT2 | 1.402794869 | 1.117263707 | 1.761297205 | 0.003558141 |
| PCAT6 | 1.097034053 | 1.021944467 | 1.177641009 | 0.010466859 |
| ZFPM2-AS1 | 1.099833794 | 1.059055439 | 1.142182298 | 7.95E-07 |
| FOXD2-AS1 | 1.175251474 | 1.083332516 | 1.274969601 | 0.000101793 |
| MYLK-AS1 | 1.275519472 | 1.047071688 | 1.553809487 | 0.015658956 |
| SLC30A6-DT | 3.324122436 | 1.606183407 | 6.879531886 | 0.00120849 |
| ZNF337-AS1 | 2.73129028 | 1.511871904 | 4.934245138 | 0.000869125 |
| HEATR6-DT | 1.30574875 | 1.004621836 | 1.697135915 | 0.046104265 |
| PRRT3-AS1 | 1.098636197 | 1.044382355 | 1.155708432 | 0.00027203 |
| PXN-AS1 | 1.19104549 | 1.046708486 | 1.355286001 | 0.007987842 |
| DNAJC9-AS1 | 2.705892979 | 1.654414777 | 4.425647616 | 7.32E-05 |
| ZNRD2-AS1 | 1.802060047 | 1.020418354 | 3.182440221 | 0.042394905 |
| MELTF-AS1 | 1.16310496 | 1.045493622 | 1.293946819 | 0.005470849 |
| SBF2-AS1 | 1.466436769 | 1.168307617 | 1.840642624 | 0.000961976 |
| SNHG12 | 1.144726733 | 1.045235343 | 1.253688274 | 0.003572298 |
| SNHG4 | 1.485983144 | 1.279163062 | 1.726242704 | 2.22E-07 |
| MYG1-AS1 | 1.475288261 | 1.161840258 | 1.873300083 | 0.001418348 |
| LINC01004 | 1.298067198 | 1.000460118 | 1.684203518 | 0.049596676 |
| HCG15 | 2.325228478 | 1.357253041 | 3.983551565 | 0.00212596 |
| C2orf49-DT | 2.598096301 | 1.393903875 | 4.842589587 | 0.002652905 |
| ASH1L-AS1 | 1.262652096 | 1.010526079 | 1.577683493 | 0.040159843 |
| DANCR | 1.019956997 | 1.005667309 | 1.034449731 | 0.006050871 |
| SNHG10 | 1.350752909 | 1.17146962 | 1.557473955 | 3.50E-05 |
| LINC02313 | 1.122399269 | 1.00614885 | 1.25208126 | 0.038466527 |
| LINC01011 | 2.026211148 | 1.331488346 | 3.083415359 | 0.000979301 |
| LNCSRLR | 2.365943309 | 1.664523612 | 3.36293682 | 1.59E-06 |
| ZBTB11-AS1 | 2.290394826 | 1.436037888 | 3.653043211 | 0.000502702 |
| LINC02561 | 1.450315124 | 1.187545723 | 1.771227766 | 0.000267026 |
| FAM111A-DT | 1.592828145 | 1.15912059 | 2.188815833 | 0.004098314 |
| MED8-AS1 | 2.638414189 | 1.631964115 | 4.265553005 | 7.55E-05 |
| EIF2AK3-DT | 2.378154729 | 1.330830187 | 4.249693141 | 0.003445727 |
| RHPN1-AS1 | 1.748170443 | 1.29659833 | 2.357013598 | 0.000248698 |
| ZEB1-AS1 | 1.479355819 | 1.189268917 | 1.840200823 | 0.000437309 |
| SNHG30 | 1.087766291 | 1.032900744 | 1.145546181 | 0.001443275 |
| NCK1-DT | 1.588407734 | 1.267123638 | 1.991154654 | 5.99E-05 |
| ARIH2OS | 1.491809181 | 1.110168991 | 2.004644924 | 0.007972885 |
| LINC00653 | 1.798738728 | 1.013874885 | 3.191183703 | 0.044741992 |
| NQO1-DT | 1.354975272 | 1.041274799 | 1.763182964 | 0.023759701 |
| HMGN3-AS1 | 2.295671316 | 1.541002402 | 3.419921205 | 4.38E-05 |
| RAB30-DT | 1.166971169 | 1.032962422 | 1.318365199 | 0.013099384 |
| ZNF232-AS1 | 1.076541167 | 1.003594402 | 1.154790105 | 0.039380548 |
| OTUD6B-AS1 | 1.114522186 | 1.01487919 | 1.223948343 | 0.023265269 |
| PSPC1-AS2 | 1.867298063 | 1.076869759 | 3.237905074 | 0.026170759 |
| LINC01275 | 1.877836578 | 1.259716401 | 2.799257206 | 0.001978379 |
| ZNF32-AS2 | 1.856634328 | 1.111718224 | 3.100687702 | 0.018044575 |
| GAS5 | 1.005388198 | 1.000477433 | 1.010323067 | 0.03147399 |
| SNHG1 | 1.046342061 | 1.019147292 | 1.074262491 | 0.000747415 |
| SREBF2-AS1 | 1.500209024 | 1.199736224 | 1.875934953 | 0.0003753 |
| SNHG3 | 1.07426194 | 1.044165904 | 1.105225436 | 7.77E-07 |
| CAPN10-DT | 1.753169039 | 1.089807142 | 2.820317064 | 0.020639927 |
| TFIP11-DT | 1.241144441 | 1.078946388 | 1.427725735 | 0.002499822 |
| MAFG-DT | 1.072939412 | 1.02707554 | 1.120851328 | 0.001585643 |
| NRAV | 1.252755636 | 1.146060295 | 1.369384044 | 6.99E-07 |
| ZNF529-AS1 | 1.321042235 | 1.058910015 | 1.64806505 | 0.013617841 |
| TGFB2-AS1 | 1.096715285 | 1.023736897 | 1.17489603 | 0.008596594 |
| CRYZL2P-SEC16B | 0.584456328 | 0.354230768 | 0.96431262 | 0.035535366 |
| PRDX6-AS1 | 1.697659286 | 1.203826609 | 2.394071565 | 0.0025472 |
| SUGT1-DT | 1.729627294 | 1.057790267 | 2.828169886 | 0.028969572 |
| ZNF32-AS1 | 1.886872953 | 1.081766931 | 3.291179864 | 0.025295419 |
| LINC00294 | 1.175643788 | 1.005958438 | 1.37395171 | 0.041884826 |
| TTC23L-AS1 | 1.546379602 | 1.066244717 | 2.242721429 | 0.021554925 |
| LINC00862 | 1.352991922 | 1.130545406 | 1.619207093 | 0.000970825 |
| SCAMP1-AS1 | 1.152741266 | 1.007125395 | 1.319411101 | 0.039111406 |
| PITPNA-AS1 | 1.064772373 | 1.006122859 | 1.126840719 | 0.029921357 |
| LINC02802 | 1.5419244 | 1.173105262 | 2.026698653 | 0.001905375 |
| LINC00942 | 1.039315632 | 1.023012361 | 1.05587872 | 1.75E-06 |
| PTOV1-AS1 | 1.282671529 | 1.086887816 | 1.513722233 | 0.003219927 |
| NIFK-AS1 | 1.296657467 | 1.085229094 | 1.549277103 | 0.004228729 |
| LINC00221 | 1.112360749 | 1.032716618 | 1.198147115 | 0.004965324 |
| MKLN1-AS | 3.487361879 | 2.328313625 | 5.223391189 | 1.36E-09 |
| KPNB1-DT | 2.989059179 | 1.32575008 | 6.739184793 | 0.008296273 |
| LINC01224 | 1.994645846 | 1.508589967 | 2.637305124 | 1.26E-06 |
| LYRM4-AS1 | 1.418474854 | 1.130790502 | 1.779348968 | 0.002504304 |
| SNHG20 | 1.282347777 | 1.065572011 | 1.54322355 | 0.008483995 |
| KTN1-AS1 | 1.873112988 | 1.080276803 | 3.24782709 | 0.025421214 |
| RNASEH1-AS1 | 1.122184571 | 1.01737781 | 1.237788165 | 0.021202602 |
| LINC01063 | 1.500823269 | 1.198058973 | 1.880099841 | 0.000412611 |
| LINC00205 | 1.253888513 | 1.116062967 | 1.408734498 | 0.000139955 |
| KDM4A-AS1 | 3.094555952 | 2.03927527 | 4.695921479 | 1.10E-07 |
| LINC00665 | 1.134202226 | 1.052581183 | 1.222152466 | 0.000950383 |
| MIR210HG | 1.157175661 | 1.082697327 | 1.23677733 | 1.70E-05 |
| SNHG21 | 1.803518143 | 1.313281799 | 2.476755328 | 0.000268584 |
| POLH-AS1 | 2.783807087 | 1.839185741 | 4.213593942 | 1.29E-06 |
| SNHG14 | 1.300891379 | 1.094834052 | 1.545730493 | 0.002792333 |
| PRKACB-DT | 1.490151718 | 1.132096995 | 1.961450433 | 0.004443044 |
| NAV2-AS6 | 1.228467544 | 1.02124846 | 1.477732958 | 0.029033463 |
| LINC00839 | 1.113533999 | 1.018136022 | 1.217870638 | 0.018608599 |
| LENG8-AS1 | 1.284934293 | 1.041937768 | 1.584601487 | 0.019074273 |
| GSEC | 1.749741747 | 1.350432181 | 2.267123241 | 2.31E-05 |
| GIHCG | 1.112760067 | 1.022113691 | 1.211445438 | 0.013720926 |
| LINC02870 | 1.090421442 | 1.038656316 | 1.144766467 | 0.000485933 |
| LUCAT1 | 1.184044908 | 1.097542035 | 1.277365512 | 1.27E-05 |
| DGUOK-AS1 | 1.508101705 | 1.065056162 | 2.13544678 | 0.02060639 |
