# Supplementary material for: Construction and Validation of a Ferroptosis-Related lncRNA Signature as a Novel Biomarker for Prognosis, Immunotherapy and Targeted Therapy in Hepatocellular Carcinoma
Source: Front Cell Dev Biol. 2022 Feb 22;10:792676. doi: 10.3389/fcell.2022.792676 (PMC8919262; doi:10.3389/fcell.2022.792676)
Supplement: Supplementary file 4 [file Table4.DOCX]

| id | coef | HR | HR.95L | HR.95H | pvalue |
| --- | --- | --- | --- | --- | --- |
| PRRT3-AS1 | 0.101132448 | 1.106423176 | 1.021424077 | 1.198495582 | 0.013148361 |
| LNCSRLR | 0.768820209 | 2.157219683 | 1.221799143 | 3.808806699 | 0.008034679 |
| MKLN1-AS | 0.731533441 | 2.078265062 | 1.132028512 | 3.81543894 | 0.018272776 |
| LINC01224 | 0.523997407 | 1.688764855 | 1.118063446 | 2.550773613 | 0.012761954 |
| LINC01063 | 0.369659239 | 1.447241368 | 1.136278573 | 1.84330465 | 0.002743477 |
| POLH-AS1 | 1.064062977 | 2.898122106 | 1.648937221 | 5.093651614 | 0.000217146 |
